# Formation of an exceptionally stable ketene during phototransformations of bicyclo[2.2.2]oct-5-en-2-ones having mixed chromophores

**DOI:** 10.3762/bjoc.16.190

**Published:** 2020-09-15

**Authors:** Asitanga Ghosh

**Affiliations:** 1 Deptartment of Chemistry, Hooghly Mohsin College, Chinsurah, Hooghly, West Bengal, 712101, India

**Keywords:** α,β- and β,γ-enones, bridgehead position, ketene, mixed chromophores

## Abstract

Photochemical reactions of bicyclo[2.2.2]oct-5-en-2-ones having mixed chromophores like a 5,6 dibenzoyl moiety and bulky electron-deficient substituents like phenyl or isopropenyl at the bridgehead position were analyzed for the first time in different solvents and upon irradiation with different wavelengths. In all cases, a regioselective photoinduced 1,5-phenyl migration leading to vinyl ketenes from the more congested site of the molecule to the less congested one has been observed. The ketenes were exceptionally stable both in air and solution. Its stability studies in acetonitrile through time-dependent UV absorption spectra revealed that it remained almost unchanged at least for a couple of weeks.

## Introduction

**Figure 1:**
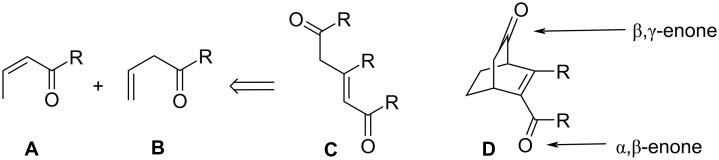
Model mixed enones.

Enones exhibit a rich and diverse photochemistry. The deep-seated photochemical rearrangements found in these systems have attracted numerous mechanistic studies. In this context, the photochemistry of α,β-enones **A** and β,γ-enones **B** (Figure 1) has become an actively researched area for more than two decades [1–6], particularly because of their fascinating photochemical rearrangements. In view of their rich photochemistry, it may be expected that incorporation of both enones into the same molecule **C** would lead to a variety of more interesting reactions. Indeed, this type of mixed enone photochemistry is also an attractive field [7–8] for chemists because the photochemistry of such substrates can feature the intramolecular competition of different photochemical rearrangements.

For several years, our laboratory (S. Lahiri et al.) is primarily engaged in synthesizing various types of such multichromophoric systems to compare their photochemistry in detail [9–15]. Since in acyclic enones like **C**, *E*/*Z*-photoisomerization becomes one of the major energy dissipation pathways, we selected rigid bicyclic multichromophoric enones like **D** to minimize this route. Interestingly such rigid systems may either be considered as α,β- or β,γ-enone with δ-keto conjugation. However, in one of our previous works [16], we have established that the system should be considered as α,β-enone with δ-keto homoconjugation.

Simple β,γ-enone systems show an absorption band at about 210–250 nm characteristic for (π,π^*^) transition and at around 280–300 nm characteristic for (n,π^*^) transition. However, enone **D** shows enhanced intensity of an (n,π^*^) absorption band near 300 nm (log ε ≈ 3.5), along with an additional weak (n,π^*^) absorption band near 350 nm (log ε ≈ 2.0). Probably this is due to interactions of these two chromophores [7]. In many cases probable unselective populations of the excited states owing to the presence of these two individual chromophores are expected to give rise to different photoproduct(s) and in some cases distribution of these photoproducts may be wavelength dependent [7].

**Scheme 1:**
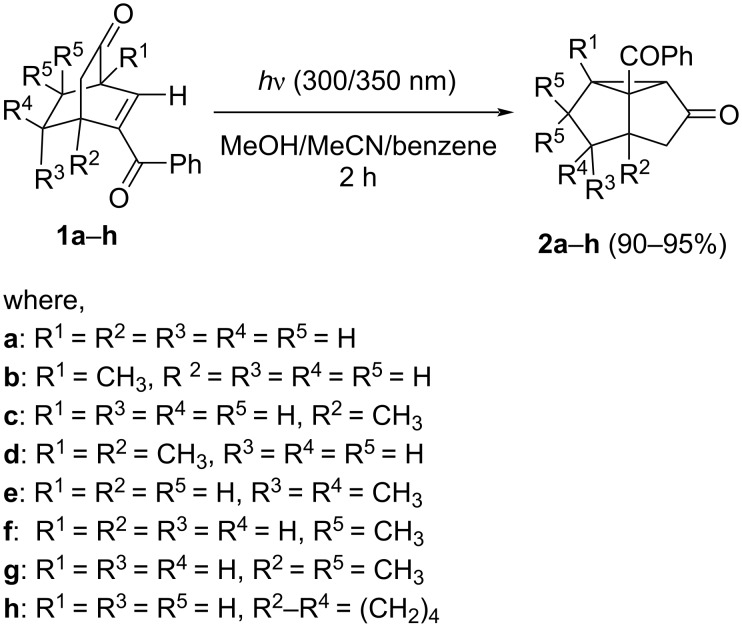
Quantitative photoisomerization of **1** to **2** in all types of solvents.

In order to investigate the photoreaction of such enones, we observed a complete 1,2-acyl shift (1,2-AS) photoproduct formation when **1a**–**h** have been irradiated (Scheme 1) [14,16]. We also observed that such a rearrangement took place very efficiently via a triplet-mediated pathway without use of any external sensitizer. Similar to bicyclic systems, some of the tricyclic system like **1h** yielded 1,2-AS product **2h** under similar reaction conditions [16].

**Scheme 2:**
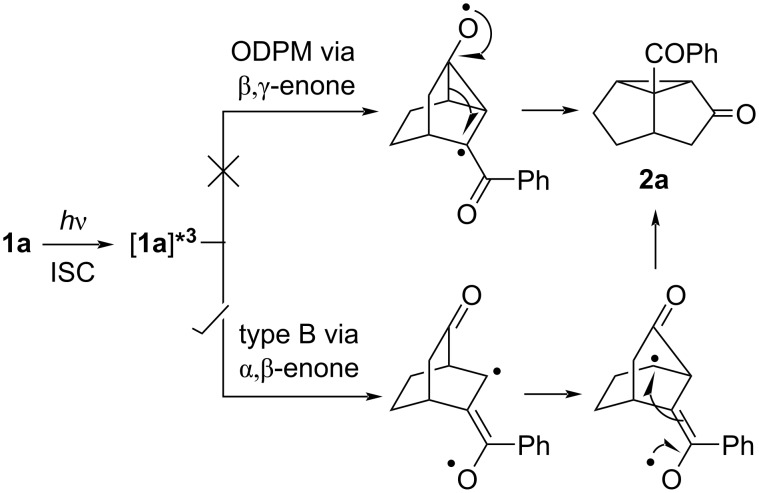
Accepted mechanistic pathway for the photochemical transformations of **1**.

Initially we envisaged two mechanistic pathways: the oxa-di-π-methane (ODPM) path (characteristic of β,γ-enone moiety) and the type-B path (characteristic of an α,β-enone moiety). However, with the help of the photoinduced electron transfer (PET) reaction of **1a**,**c**,**d**,**g**–**h** and photoreaction of the partially olefinated product of **1c**,**d**,**h**, we have recognized that in a mixed α,β- and β,γ-enone system, the photorearrangement route is selectively from the α,β-enone part and we confirmed that the irradiation follows a type B rearrangement (Scheme 2) [16].

**Scheme 3:**
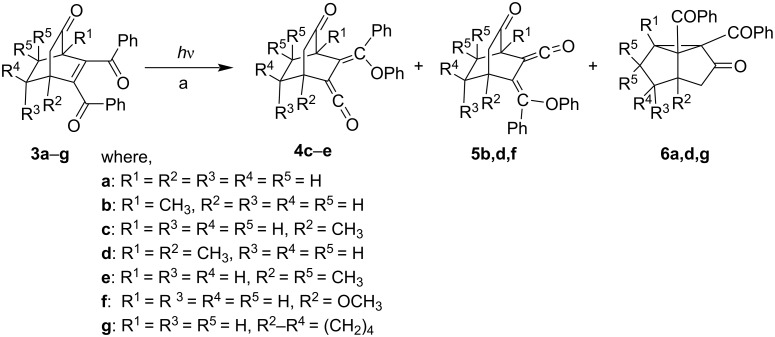
Photochemical reactions of **3a**–**g**. Irradiation using a Hanovia medium pressure 450 W lamp with a pyrex filter; a) conditions and yields are given in Table 1.

Another interesting set of competitive 1,5-(C–O) acyl shift rearrangements (formation of vinyl ketene) with that of 1,2-AS were observed when photoreactions of bridged bicyclic systems like **3a**–**g** were carried out [9–13]. These molecules contain a δ-keto-α,β-enone system juxtaposed with a (*cis*)-dibenzoylalkene moiety. Interestingly, photoreactions of **3a**–**f** were found to follow regioselective photoindiuced 1,5-phenyl migration from the more congested site of the molecule to the less one leading to the formation of vinyl ketenes **4c**–**e** and **5b,d,f** along with the formation of the 1,2-AS photoproduct **6a,d** (Scheme 3, Table 1). Some of the vinyl ketenes were stable enough to be kept at room temperature up to 24 hours together as verified by time-dependent absorption spectra [13]. Also no external sensitizer was needed for these transformations. So an efficient intersystem crossing (ISC) may be involved in such systems to reach to the triplet state. A further fascinating aspect is that the two competitive pathways viz. 1,2-AS and photoindiuced 1,5-phenyl migration have been found to be subtly dependent on the substitution pattern. For instance, the rate of 1,2-AS in **3b** has been found to be much faster than the one of **3e**. It is probably due to the extra hindrance of *gem*-dimethyl substituents at C-7 position in the latter. Moreover, only 1,2-AS photoproduct **6g** was obtained when **3g** was irradiated [10].

**Table 1:** Chemical yields of the formation of photoproducts **4**, **5** and **6**.

Starting enones	Time (h)	Medium	Photoproduct(s), yield (%)

**3a**	5	acetone	**6a** (25)^a^
**3b**	≈35	suspended in distilled water	**5b** (55)^b^
**3c**	≈35	suspended in distilled water	**4c** (55)^b^
**3d**	6	acetone	**4d** (13)^a^, **5d** (17)^a^, **6d** (23)^a^
**3e**	≈35	suspended in distilled water	**4e** (82)^b^
**3f**	≈35	suspended in distilled water	**5f** (85)^b^
**3g**	5 5.5 5	acetone benzene MeOH	**6g** (60)^a^ **6g** (45)^a^ **6g** (55)^a^

^a^Isolated yields; ^b^based on recovered starting materials.

**Figure 2:**
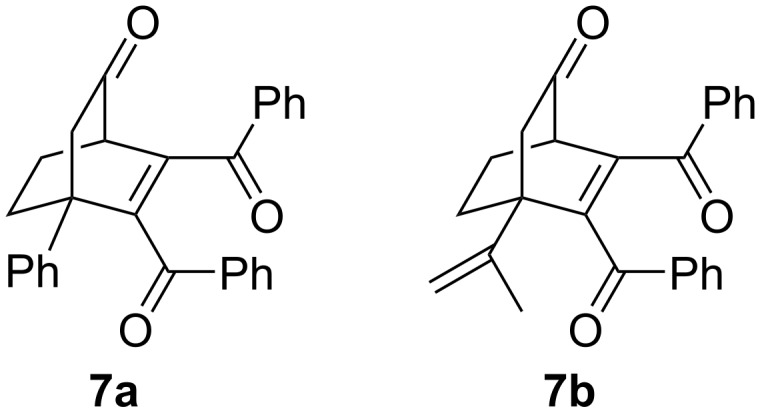
Enones used for this work.

In all of the molecules, the bridgehead positions contain only electron-donor groups (such as OMe, Me, etc.), but same type of rigid enones having electron deficient groups at the bridgehead positions have not been checked so far. To bridge this gap, we carried out the photoreactions of 5,6-dibenzoyl-4-phenylbicyclo[2.2.2]oct-5-en-2-one (**7a**) and 5,6-dibenzoyl-4-isopropenylbicyclo-[2.2.2]oct-5-en-2-one (**7b**) in detail (Figure 2). Both of the molecules contain electron-acceptor groups like phenyl and isopropenyl at its bridgehead position (C-4) and these groups were bulkier than the earlier reported one.

## Results and Discussion

**Scheme 4:**
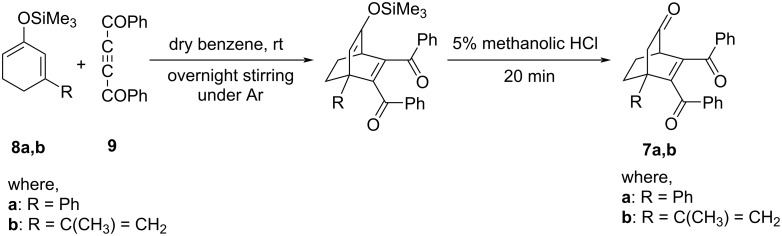
Synthesis of **7a**,**b**.

5,6-Dibenzoyl-4-phenylbicyclo[2.2.2]oct-5-en-2-one (**7a**) and 5,6-dibenzoyl-4-isopropenylbicyclo[2.2.2]oct-5-en-2-one (**7b**) have been prepared through Diels–Alder reaction between substituted silyloxycyclohexa-1,3-dienes **8a** and **8b** [17–18] and dibenzoyl acetylene (**9**) followed by hydrolysis of the silyloxy adduct (Scheme 4).

Both **7a** and **7b** showed an enhanced (n,π*) absorption band near 300 nm (log ε ≈ 3.5) along with an additional weak band near 343 nm (log ε ≈ 2.5) in their UV spectra as expected for δ-keto-α,β-enone [7].

### Photoreaction of **7a** and **7b**

**Scheme 5:**
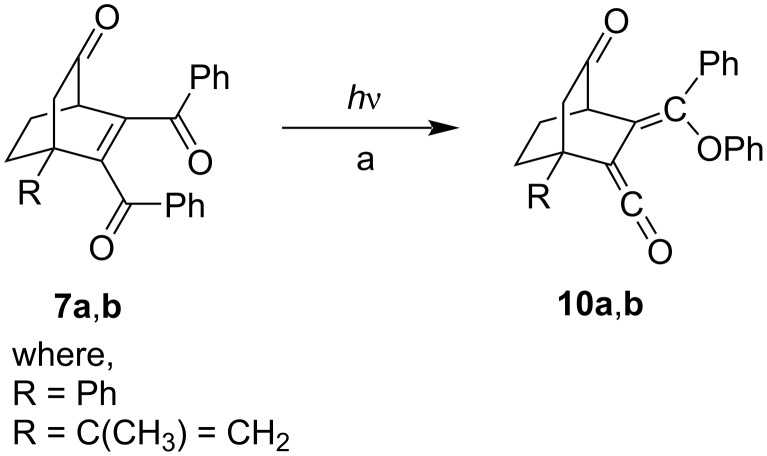
Photochemical reaction of **7a**,**b**; a) solvent and conditions are given in Table 2.

Irradiation of a degassed solution of **7a**,**b** in benzene at 254 nm for 3–4 h yielded a pale yellow solid of ketene **10a**,**b** (Scheme 5, Table 2) through regioselective photoinduced 1,5-phenyl migration and we did not obtain any 1,2-AS or any other photoproduct. The same ketenes were isolated in moderate yields in acetone and acetonitrile solvents. However, we did not find an alternative 1,2-AS photoproduct. Even a change of the wavelength had no effect on the reactivity and similar results were obtained at 300 and 350 nm. The results are summarized in Table 2.

**Table 2:** Result of photoreaction of **7a**,**b** at various wavelengths in different solvents.

Wavelength (nm)	Solvent	Starting compound^a^	Irradiation time (h)	Product^b^ (yield/ %)

254	benzene	**7a**	3	**10a** (38)
		**7b**	4	**10b** (32)
	acetonitrile	**7a**	3	**10a** (54)
		**7b**	4	**10b** (38)
	acetone	**7a**	3	**10a** (44)
		**7b**	4	**10b** (33)
300	benzene	**7a**	5	**10a** (46)
		**7b**	5.5	**10b** (41)
	acetonitrile	**7a**	5	**10a** (41)
		**7b**	5.5	**10b** (40)
	acetone	**7a**	5	**10a** (49)
350	benzene	**7a**	5	**10a** (43)
	acetonitrile	**7a**	5	**10a** (49)

^a^Prepared for the first time, ^b^isolated yield.

The structures of ketenes **10a**,**b** were established from their analytical and spectral data. The ketene stetching (γ_C=C=O_) was detected in the IR spectrum near 2090 cm^−1^ and the ring ketone stetching (γ_C=O_) appeared at 1730 cm^−1^. The ^1^H NMR spectra of these compounds were very similar to those of the parent molecules but their ^13^C NMR data were found to be much more informative (Table 3). In ^1^H NMR, a doublet and a triplet in the region of δ 6.6–6.9 ppm confirmed the presence of the OPh group while the bridgehead protons of the ketenes appeared at a slightly up-field position with respect to the parent compounds.

**Table 3:** Comparison of spectral data of ketens **10a**,**b** with parent compounds **7a**,**b**.

Compound (mp [°C])	IR [cm^−1^]		^1^H NMR [ppm]		^13^C NMR [ppm]			
	γ_C=C=O_	γ_C=O_	δ_bridgehead_	δ_OPh_	δ_C+O_	δ_+C+O_	δ_C+C+O_	δ_+C-OPh_

**7a** (224–226)	–	1726 1666 1646	3.82 (t, *J* = 2.5 Hz)	–	208.4, 195.4, 193.3	–	–	–
**7b** (144–146)	–	1722 1663 1649	3.73 (t, *J* = 2.7 Hz)	–	208.9, 194.8, 193.6	–	–	–
**10a** (162–164)	2087	1730	3.69 (brs)	6.68 (d, *J =* 8 Hz, 2H), 6.79 (t, *J =* 7.4 Hz, 3H)	209.9	200.2	48.9	155.9
**10b** (128–130)	2094	1732	3.67 (brs)	6.77 (d, *J =* 8 Hz, 2H), 6.91 (t, *J =* 7.3 Hz, 3H)	210.3	204.6	48.9	144.3

**Figure 3:**
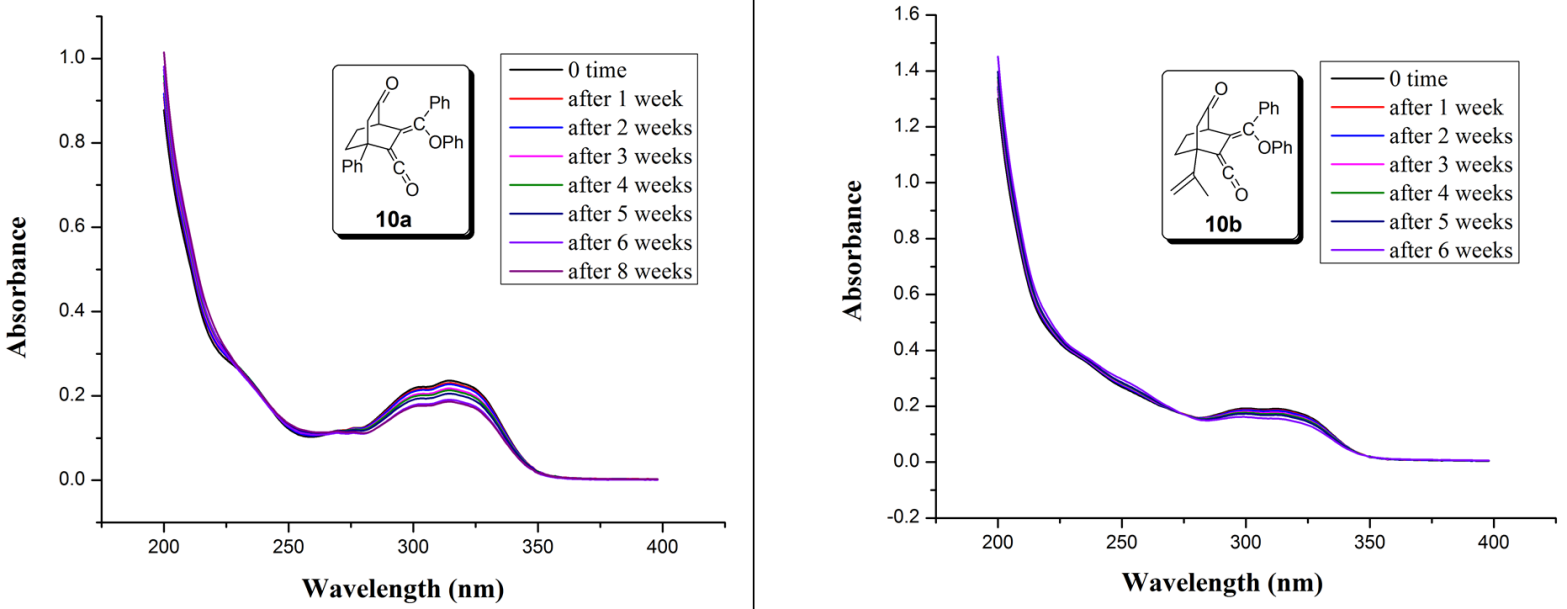
Time-dependent absorption spectra of **10a**,**b** in acetonitrile at rt.

Ketenes **10a**,**b** were exceptionally stable both in air and solution. Their stability studies in acetonitrile through time-dependent absorption spectra (Figure 3) revealed that they remained almost unchanged at least for six to eight weeks.

Such photoinduced intramolecular 1,5-phenyl migration from carbon to proximate oxygen is a general reaction route for (*cis*)-dibenzoylalkene chromophores [19–22]. In unsymmetrical derivatives such migrations have been found to take place from the benzoyl group attached to the more crowded olefinic carbon atom to the less crowded one [23]. A similar observation has been reported from our laboratory for bicyclo[2.2.2]octenone systems [13]. Thus, selective formation of **10a**,**b** is expected with the exception that the ketenes **10a**,**b** were unusually stable. This may be due to the imposition of the bulky electron-withdrawing group at the bridgehead position. The non-requirement of any sensitizer in the process indicates that the reaction presumably proceeds via a triplet-mediated pathway. Moreover, the non-appearence of any 1,2-AS product signifies that the β,γ-enone part is inactive. On the other hand (*cis*)-dibenzoylalkene like α,β-enone part of such multichromophoric bicyclic system actively participates during the photoirradiation.

**Scheme 6:**
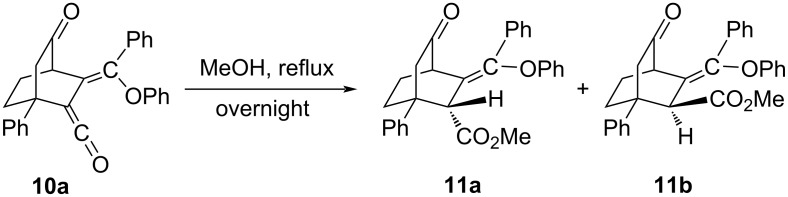
Conversion of ketene **10a** to its methyl esters **11a**,**b**.

The structure of **10a** was further confirmed by converting it quantitatively to the corresponding methyl esters **11a**,**b** with methanol under reflux conditions (Scheme 6). The isomeric ratio (1:6) of **11a** and **11b** was confirmed from the NMR spectrum of the crude reaction mixture. Since the polarity of the two methyl esters is very similar, we could not separate them through column chromatography. However, **11a** was separated in a trace amount through repeated preparative TLC.

Moreover, when a degassed solution of **7a** in methanol was irradiated under similar reaction conditions, a mixture of methyl esters **11a** and **11b** was obtained in the same 1:6 ratio with a moderate yield (60%).

## Conclusion

From the above results it was evident that the presence of phenyl or isopropenyl like electron-deficient bulky substituents at the bridgehead position of bicyclo[2.2.2]oct-5-en-2-ones juxtaposed with the (*cis*)-dibenzoylalkene moiety facilitates the regioselective photo-1,5-phenyl migration from the more congested site of the molecule with the formation of vinyl ketenes presumably via a triplet-mediated pathway. To the best of our knowledge this is the first example of the formation of ketenes which are exceptionally stable for more than 6–8 weeks. Furthermore, we conclude that in a mixed α,β- and β,γ-enone type system, the photorearrangement occures selectively from the α,β-enone part since there was no 1,2-AS or ODPM rearranged photochemical product.

## Experimental

The synthetic procedures, photochemical studies and spectra (UV, IR, mass, ^1^H NMR, ^13^C-NMR, DEPT-135) of all new compounds can be found in the Supporting Information File 1 and Supporting Information File 2.

## Supporting Information

File 1 Experimental procedures and analytical data.

File 2 Photochemical studies and spectra (UV, IR, mass, ^1^H NMR, ^13^C NMR, DEPT-135) of all new compounds.
