# Supplementary material for: Formation of an exceptionally stable ketene during phototransformations of bicyclo[2.2.2]oct-5-en-2-ones having mixed chromophores
Source: Beilstein J Org Chem. 2020 Sep 15;16:2297–303. doi: 10.3762/bjoc.16.190 (PMC7509375; doi:10.3762/bjoc.16.190)
Supplement: File 1 — Experimental procedures and analytical data. [file Beilstein_J_Org_Chem-16-2297-s001.pdf]

BEILSTEIN JOURNAL OF ORGANIC CHEMISTRY

Supporting Information

for

Formation of an exceptionally stable ketene during phototransformations of bicyclo[2.2.2]oct-5-en-2-ones having mixed chromophores

Asitanga Ghosh

Beilstein J. Org. Chem. **2020,** *16,* 2297–2303. doi:10.3762/bjoc.16.190

Experimental procedures and analytical data

License and Terms: This is a supporting information file under the terms of the Creative Commons Attribution License (https://creativecommons.org/licenses/by/4.0). Please note that the reuse, redistribution and reproduction in particular requires that the authors and source are credited.
The license is subject to the *Beilstein Journal of Organic Chemistry* terms and conditions: (https://www.beilstein-journals.org/bjoc)

1. Experimental procedures

1-1. General experimental information

The melting points were uncorrected and were measured in a Gallenkamp melting point apparatus. The IR spectra were recorded on a FTIR-8300 Shimadzu spectrometer and/or on a Perkin-Elmer Spectrum Two FT-IR spectrometer. All NMR spectra were recorded in $CDCl_3$ solution at 300 MHz for ^{1}H and at 75 MHz for ^{13}C on a Brucker AC-300 spectrometer using tetramethylsilane as an internal standard. High resolution mass spectra were recorded on a Qtof Micro YA263 spectrometer using the electron spray ionization (ESI) technique. UV absorption spectra were recorded on a Shimadzu UV-2401PC spectrophotometer. Flash column chromatography was performed using silica gel (230–400 mesh) under medium pressure and ordinary column chromatography was performed using silica gel (60–120 mesh). Preparative thin layer chromatography was performed using silica gel GF-254 (Merck). Petroleum ether and ethyl acetate have been abbreviated as PE and EA. PE used with a boiling range of 60–80 °C. Spectral grade solvents (Merck) were used for recording UV spectra. Solvents were purified and dried before using. Irradiation experiments were carried out in a Southern New England Rayonet Photochemical Reactor using lamps of the desired wavelength.

Dibenzoyl acetylene (**9**, DBA) [1] was prepared according to the literature procedure.

1-2. Synthesis of 5,6-dibenzoyl-4-phenylbicyclo[2.2.2]oct-5-en-2-one (7a) The trimethyl(5-phenylcyclohexa-1,5-dienyloxy)silane (**8a**, 3.66 gm, 15 mmol) was prepared from 3-phenylcyclohexenone [2] following our previously reported procedure [3,4]. As also described in [3] dry benzene (3 mL) and a solution of DBA (**9**, 2.34 gm,10 mmol) in dry benzene (10 mL) were immediately added to the diene and the mixture was stirred overnight under an argon atmosphere. After that 5%

methanolic HCl (5 mL) was added to it and the mixture was stirred for additional 20 min. After removal of MeOH, the mixture was extracted with ether (3 × 25 mL). The organic phases were combined, dried over Na_2SO_4, and evaporated in vacuo. The residue was filtered through a small column with 10% EA in PE which gave the colourless solid of 5,6-dibenzoyl-4- phenyl-bicyclo[2.2.2]oct-5-en-2-one (**7a**, 3.41 g, 84%), mp 224–226 °C, as colorless crystals after recrystallization from DCM–PE (1:10) mixture. HRMS Calcd for $C_{28}H_{22}O_3$**:** $[M+Na]^+$ 429.1467; Found**:** 429.1462; *IR (neat)* λ_{max} (cm^{-1})**:** 1726 (C=O), 1666 (C=O), 1646 (C=O), 1595(C=C); UV (MeCN) λ_{max} (nm)**:** 255 (log ε 4.01), 283 (3.6), 343 (2.4); 1H-NMR δ (ppm)**:** 2.15-2.32 (m, 3H), 2.55 (d, J 18.5 Hz) superimposed with 2.50-2.59 (m) total 2H, 3.04 (dd, J 18.5 and 3.3 Hz, 1H), 3.82 (t, J 2.5 Hz, 1H), 7.12-7.49 (m, 15H); ^{13}C NMR δ (ppm)**:** 23.9 (CH_2), 31.3 (CH_2), 45.5 (CH_2), 49.1(C), 51.1 (CH), 126.6 (CH), 127.2 (CH), 128.25 (CH), 128.29 (CH), 128.34 (CH), 129.1 (CH), 133.0 (CH), 133.15 (CH), 136.1 (C), 137.1 (C), 138.4 (C), 140.3 (C), 154.8 (C), 193.3 (CO), 195.4 (CO), 208.4 (CO).

1-3. Synthesis of 5,6-dibenzoyl-4-isopropenylbicyclo[2.2.2]oct-5-en-2-one (7b)

The pale yellow liquid of trimethyl(5-phenylcyclohexa-1,5-dienyloxy)silane (**8b**) (2.08 g, 10 mmol) was prepared from the 3-isopropenyl-cyclohex-2-enone [5] (1.36 g, 10.0 mmol) as reported earlier [3,4]. As also described in [3] dry benzene (5 mL) and a solution of DBA (1.638 gm, 7 mmol) in dry benzene (5 mL) were immediately added to the diene and the mixture was stirred for 6 h under inert atmosphere. A solution of 5% methanolic HCl (5 mL) was added to it and the mixture was stirred for additional 20 min. After removal of MeOH the mixture was extracted with ether (3 × 25 mL). The organic phases were combined, dried over anhydrous Na_2SO_4, and evaporated in vacuo. The residue was chromatographed over a silica gel column. Elution of the

column with 15% EA in PE gave a solid of 5,6-dibenzoyl-4-isopropenyl-bicyclo[2.2.2]oct-5-en-2-one (**7b**, 1.09 g, 43%), mp 144–146 °C, as colorless crystals after recrystallization from DCM–PE (1:10) mixture. HRMS Calcd for $C_{25}H_{23}O_3$**:** $[M+H]^+$ 371.1321, found**:** 371.1327. IR (neat) ν_{max} (cm^{-1}): 1722 (C=O), 1663 (C=O), 1649 (C=O), 1595 (C=C). UV (MeCN) λ_{max} (nm)**:** 256 (log ε 3.7), 284 (3.4), 346 (2.3). ^{1}H NMR δ (ppm)**:** 1.64 (s, 3H), 1.91-1.99 (m, 1H), 2.06-2.22 (m, 2H), 2.29 (d, *J* 18.5 Hz) superimposed with 2.26-2.35 M total 2H, 2.79 (dd, *J* 18.5 and 2.9 Hz, 1H), 3.73 (t, *J* 2.7 Hz, 1H), 4.96 (s, 1H), 4.98 (s, 1H), 7.24-7.51(m, 10H). ^{13}C NMR δ (ppm)**:** 21.6 (CH_3), 24 (CH_2), 29.9(CH_2), 44.2(CH_2), 50.9(C), 51.1(CH), 114.5 (=CH_2), 128.5 (CH), 128.6 (CH), 128.8 (CH), 129.2 (CH), 133.3 (CH), 133.5 (CH), 136.5(C), 137.1 (C), 139.2 (C), 44.1 (C), 152.7 (C), 193.6 (CO), 194.8 (CO), 208.9 (CO).

1-4. Photolysis of 5,6-dibenzoyl-4-phenylbicyclo[2.2.2]oct-5-en-2-one (7a)

a. **at 254 nm:**

i. In benzene: A 100 mL solution of **7a** (380 mg, 0.94 mmol) in dry benzene was degassed with argon for 10 minutes and then irradiated at 254 nm for 3h in a quartz tube. Solvent was removed under reduced pressure and the residue was subjected to flash chromatography. Elution of the column with 8% EA in PE gave a yellow solid of vinyl ketene (*Z*)-5-formyl-6-(phenoxy(phenyl)methylene)-4-phenylbicyclo[2.2.2]octan-2-one (**10a**, 144 mg, 38%), m.p. 162–164 °C. HRMS for $C_{28}H_{22}O_3Na$ calcd. 429.1467; found ($[M+Na]^+$): 429.1463. UV (MeCN) λ_{max}(nm): 227 (log ε 3.64), 316 (3.59). IR (neat) ν_{max} (cm^{-1}): 2087 (=C=C=O), 1730 (C=O), 1591(C=C). ^{1}H-NMR δ (ppm): 1.95-2.18 (m, 3H), 2.46-2.59 (m) superimposed with 2.56 (d, *J* 18.2 Hz) total 2H, 3.01 (dd, *J* 18.2 and 3 Hz, 1H), 3.69 (brs, 1H), 6.68 (d, *J* 8 Hz, 2H), 6.79 (t, *J* 7.4 Hz, 3H), 7.02 -7.03 (m, 10H). ^{13}C NMR δ (ppm): 24.3 (CH_2),

32.6 (CH_2), 41.9 (C), 46.9 (CH), 48.9 (C=C=O), 51.0 (CH_2), 116.5 (CH), 117.4 (C), 122.5 (CH), 126.3 (CH), 128.1 (CH), 128.2 (CH), 128.7 (CH), 128.9 (CH), 129.6 (CH), 129.8 (CH), 133.2 (C), 140.9 (C), 141.3 (C), 155.9 (C(Ph)OPh), 200.2 (CO), 209.9 (CO).

Further elution with same solvent gave a white solid of unreacted starting material 5,6-dibenzoyl-4-phenylbicyclo[2.2.2]oct-5-en-2-one (**7a**, 185 mg, 49%),

ii. Trapping of the ketene 10a: The ketene **10a** (150 mg) was refluxed in dry methanol for overnight. Solvent was removed and the reaction mixture was found to contain two isomeric esters (2*R*,*Z*)-methyl 5-oxo-3-(phenoxy(phenyl)methylene)-1-phenylbicyclo[2.2.2]octane-2-carboxylate (**11a**) and (2*S*,*Z*)-methyl 5-oxo-3-(phenoxy(phenyl)methylene)-1-phenylbicyclo[2.2.2]octane-2-carboxylate (**11b**) in a quantitative amount. From the NMR spectrum, **11a** and **11b** were found to be in a ≈1:6 ratio in the mixture. Since, the polarity of these two isomers was almost same, it was difficult to separate them from the mixture and only 21 mg of **11a** had been isolated as a white solid (m.p. 174–176 °C) by repeated thin layer chromatography with 12% EA in PE. HRMS calcd. for $C_{29}H_{26}O_4Na$: 461.1729; found ($[M+Na]^+$): 461.1729. IR (neat) ν_{max} (cm^{-1}): 1728 (C=O), 1593 (C=C). ^{1}H NMR δ (ppm): 1.71-1.78 (m, 1H), 1.99-2.06 (m, 1H), 2.38 (d, *J* 11.4 Hz, 1H), 2.42-2.47 (m, 1H), 2.72-2.78 (m, 1H), 2.85 (dd, *J* 11.4 and 2.1 Hz, 1H), 3.50 (t, *J* 3 Hz, 1H), 3.77 (s, 1H), 6.65 (d, *J* 4.8 Hz, 2H), 6.77 (t, *J* 4.2 Hz, 3H), 7.01-7.05 (m, 2H), 7.15-7.32 (m, 10H). ^{13}C NMR δ (ppm): 23.6 (CH_2), 24.9 (CH_2), 43.0 (C), 46.9 (CH_3), 51.3 (CH), 51.4 (CH_2), 53.2 (CH), 111.9 (C), 116.8 (CH), 120.6 (CH), 122.2 (CH), 125.9 (CH), 127.3 (CH), 128.57 (CH), 128.63 (CH), 128.9 (CH), 129.3 (CH), 129.4 (CH), 133.0 (C), 142.7 (C), 148.4 (C), 155.5 (C), 172.4 (CO_2Me), 210.3 (CO).

ii. In acetonitrile: A 100 mL solution of the **7a** (380 mg, 0.94 mmol) in dry acetonitrile was degassed with argon for 10 minutes and then irradiated for 3 h in a quartz tube. The solvent was removed under reduced pressure and the residue was subjected to flash chromatography. Elution of the column with 8% EA in PE gave a yellow solid of **10a** (205 mg, 54%), m.p. 162-164 ^{0}C (mmp 164 ^{0}C). Further elution with same solvent gave a white solid of unreacted starting material **7a** (132 mg, 35%), m.p. 225 °C (mmp 224–226 °C).

iii. In acetone: A 100 mL solution of the **7a** (380 mg, 0.94 mmol) in dry acetone was degassed with argon for 10 minutes and then irradiated for 3 h in a quartz tube. Solvent was removed under reduced pressure and the residue was subjected to flash chromatography. Elution of the column with 8% EA in PE gave a yellow solid of **10a** (167mg, 44%), m.p. 162–164 °C (mmp 164 °C). Further elution with same solvent gave a white solid of unreacted starting material **7a** (161 mg, 42%), m.p. 225 °C (mmp 224–226 °C).

iv. In methanol: A 50 mL solution of **7a** (110 mg, 0.5 mmol) in dry methanol was degassed with argon for 10 minutes and then irradiated for 4 h in a quartz tube. Solvent was removed under reduced pressure and the residue was chromatographed over silica gel. Elution of the column with 10% EA in PE gave a mixture of two isomeric esters **11a** and **11b** (71 mg, 60%). From the NMR spectrum, **11a** and **11b** were found to be in a ≈1:6 ratio in the mixture.

b. at 300 nm:

i. In benzene: A 60 mL solution of **7a** (240 mg, 0.59 mmol) in dry benzene was degassed with argon for 10 minutes and then irradiated for 5 h in a Pyrex tube. The

solvent was removed under reduced pressure and the residue was subjected to flash chromatography. Elution of the column with 8% EA in PE gave a yellow solid of **10a** (110 mg, 46%), m.p. 162–164 °C (mmp 164 °C). Further elution with same solvent gave a white solid of unreacted starting material **7a** (82 mg, 34%), m.p. 225 °C (mmp 224–226 °C).

ii. In acetonitrile: A 60 mL solution of **7a** (240 mg, 0.59 mmol) in dry acetonitrile was degassed with argon for 10 minutes and then irradiated for 5 h in a Pyrex tube. Solvent was removed under reduced pressure and the residue was subjected to flash chromatography. Elution of the column with 8% EA in PE gave a yellow solid of **10a** (98 mg, 41%), m.p. 162–164 °C (mmp 164 °C). Further elution with same solvent gave a white solid of unreacted starting material **7a** (101 mg, 42%), m.p. 225 °C (mmp 224–226 °C).

iii. In acetone: A 70 mL solution of **7a** (200 mg, 0.5 mmol) in dry acetone was degassed with argon for 10 minutes and then irradiated for 5 h in a pyrex tube. Solvent was removed under reduced pressure and the residue was subjected to flash chromatography. Elution of the column with 8% EA in PE gave a yellow solid of **10a** (98 mg, 49%), m.p. 162–164 °C (mmp 164 °C). Further elution with same solvent gave a white solid of unreacted starting material **7a** (80 mg, 40%), m.p. 225 °C (mmp 224–226 °C).

c. at 350 nm:

i. In benzene: A 70 mL solution of **7a** (180 mg, 0.45 mmol) in dry benzene was degassed with argon for 10 minutes and then irradiated for 5 h in a Pyrex tube.

Solvent was removed under reduced pressure and the residue was subjected to flash chromatography. Elution of the column with 8% EA in PE gave a yellow solid of **10a** (77 mg, 43%), m.p. 162–164 °C (mmp 164 °C). Further elution with same solvent gave a white solid of unreacted starting material **7a** (83 mg, 46%), m.p. 225 °C (mmp 224–226 °C).

ii. In acetonitrile: A 70 mL solution of **7a** (180 mg, 0.45 mmol) in acetonitrile was degassed with argon for 10 minutes and then irradiated for 5 h in a Pyrex tube. Solvent was removed under reduced pressure and the residue was subjected to flash chromatography. Elution of the column with 8% EA in PE gave a yellow solid of 334 (88 mg, 49%), m.p. 162–164 °C (mmp 164 °C). Further elution with same solvent gave a white solid of unreacted starting material **10a** (70 mg, 39%), m.p. 225 °C (mmp 224–226 °C).

1-5. Photolysis of 5,6-dibenzoyl-4-isopropenylbicyclo[2.2.2]oct-5-en-2-one (7b)

a. at 254 nm:

i. In benzene: A 50 mL solution of **7b** (185 mg, 0.5 mmol) in dry benzene was degassed with argon for 10 minutes and then irradiated for 4 h in a quartz tube. Solvent was removed under reduced pressure and the residue was chromatographed over silica gel. Elution of the column with 5% EA in PE gave a yellow solid of vinyl ketene (*Z*)-5-formyl-6-(phenoxy(phenyl)methylene)-4-(prop-1-en-2-yl)bicyclo[2.2.2]-octan-2-one (**10b**, 60 mg, 32%), m.p. 128–130 °C. HRMS calcd. for $C_{25}H_{22}O_3K$: 409.1206; found ($[M+K]^+$): 409.1208. IR (neat) ν_{max} (cm^{-1}): 2094 (=C=C=O), 1732 (C=O), 1593 (C=C). UV (MeCN) λ_{max}(nm): 230 (log ε 3.63), 314 (3.34). ^{1}H-NMR δ (ppm) : 1.73 (s, 3H), 1.75-2.11 (m, 3H), 2.37 (d, *J* 17 Hz) superimposed with 2.29-

2.40 (m) total 2H, 2.85 (dd, *J* 17 and 2.7 Hz, 1H), 3.67 (brs, 1H), 5.01 (s, 1H), 5.04 (s, 1H), 6.77 (d, *J* 8 Hz, 2H), 6.91 (t, *J* 7.3 Hz, 3H),, 7.12-7.37 (m, 5H). ^{13}C NMR δ (ppm): 20.2 (CH_3), 24.2 (CH_2), 30.6 (CH_2), 43.4 (C), 46.6 (CH), 48.9 (C=C=O), 49.2 (CH_2), 114.3 (=CH_2), 116.5 (CH), 122.5 (CH), 125.8 (C), 125.9 (C), 128.1 (CH), 128.5 (CH), 128.6 (CH), 129.2 (C), 129.6 (CH), 133.2 (C), 144.3 (C(Ph)OPh), 204.6 (CO), 210.3 (CO). Further elution with 12% EA in PE gave a white crystalline solid of unreacted starting material **7b** (91 mg, 49%), m.p. 145 °C (mmp 144–146 °C).

ii. In acetonitrile: A 50 mL solution of **7b** (185 mg, 0.5 mmol) in dry acetonitrile was degassed with argon for 10 minutes and then irradiated for 4h in a quartz tube. Solvent was removed under reduced pressure and the residue was chromatographed over silica gel. Elution of the column with 5% EA in PE gave a yellow solid of **10b** (71 mg, 38%, mmp 130 °C). Further elution with 12% EA in PE gave a white crystalline solid of unreacted starting material **7b** (76 mg, 41%), m.p. 145 °C (mmp 144–146 °C).

iii. In acetone: A 50 mL solution of the **7b** (185 mg, 0.5 mmol) in dry acetonitrile was degassed with argon for 10 minutes and then irradiated for 4h in a quartz tube. Solvent was removed under reduced pressure and the residue was chromatographed over silica gel. Elution of the column with 5% EA in PE gave a yellow solid of **10b** (61 mg, 33%, mmp 130 °C). Further elution with 12% EA in PE gave a white crystalline solid of unreacted starting material **7b** (87 mg, 47%), m.p. 145 °C (mmp 144–146 °C).

b. at 300 nm:

i. In Benzene: A 60 mL solution of **7b** (200 mg, 0.54 mmol) in dry benzene was degassed with argon for 10 minutes and then irradiated for 5.5 h in a quartz tube.

Solvent was removed under reduced pressure and the residue was chromatographed over silica gel. Elution of the column with 5% EA in PE gave a yellow solid of **10b** (82 mg, 41%, mmp 130 ºC). Further elution with 12% EA in PE gave a white crystalline solid of unreacted starting material **7b** (102 mg, 51%), m.p. 145 °C (mmp 144–146 °C).

ii. In acetonitrile: A 60 mL solution of the **7b** (200 mg, 0.54 mmol) in acetonitrile was degassed with argon for 10 minutes and then irradiated for 5.5 h in a quartz tube. Solvent was removed under reduced pressure and the residue was chromatographed over silica gel. Elution of the column with 5% EA in PE gave a yellow solid of **10b** (80 mg, 40%, mmp 130 °C). Further elution with 12% EA in PE gave a white crystalline solid of unreacted starting material **7b** (94 mg, 47%, m.p. 145 °C (mmp 144–146 °C).

1-6. References

1. Lutz, R. E.; Smithey, W. R. *J. Org. Chem.* **1951**, *16*, 51.
2. Shawe, T. T.; Hansen, D. B.; Peet, K. A.; Prokopowicz, S. A.; Robinson, P. M.; Cannon, A.; Dougherty, K. E.; Ross, A. A.; Landino, L. M. *Tetrahedron.* **1997**, 53, 1547.
3. Ghosh, A.; Chakraborty, I.; Adarsh, N. N.; Lahiri, S. *Tetrahedron*. **2010**, *66*, 164.
4. The *O*-silyloxydienes were highly moisture sensitive, hence all of the steps of the reaction were performed following the same procedure as described in [3].
5. Barriault, L.; Thomas, J. D. O.; Cle´ment, R. *J. Org. Chem.* **2003**, *2317*.
