# Supplementary material for: Formation of an exceptionally stable ketene during phototransformations of bicyclo[2.2.2]oct-5-en-2-ones having mixed chromophores
Source: Beilstein J Org Chem. 2020 Sep 15;16:2297–303. doi: 10.3762/bjoc.16.190 (PMC7509375; doi:10.3762/bjoc.16.190)
Supplement: File 2 — Photochemical studies and spectra (UV, IR, mass, 1H NMR, 13C NMR, DEPT-135) of all new compounds. [file Beilstein_J_Org_Chem-16-2297-s002.zip › Spectral+Data-Asitanga.pdf]

Supporting Information
for

Formation of exceptionally stable ketene during photo transformations of Bicyclo[2.2.2]oct-5-en-2-ones having mixed chromophores

Asitanga Ghosh*

Dept. of Chemistry, Hooghly Mohsin College, Chinsurah, Hooghly, West Bengal, India- 712101

Copies of NMR, IR, Mass & UV spectra

2-1. NMR, IR, Mass and UV Spectrum:

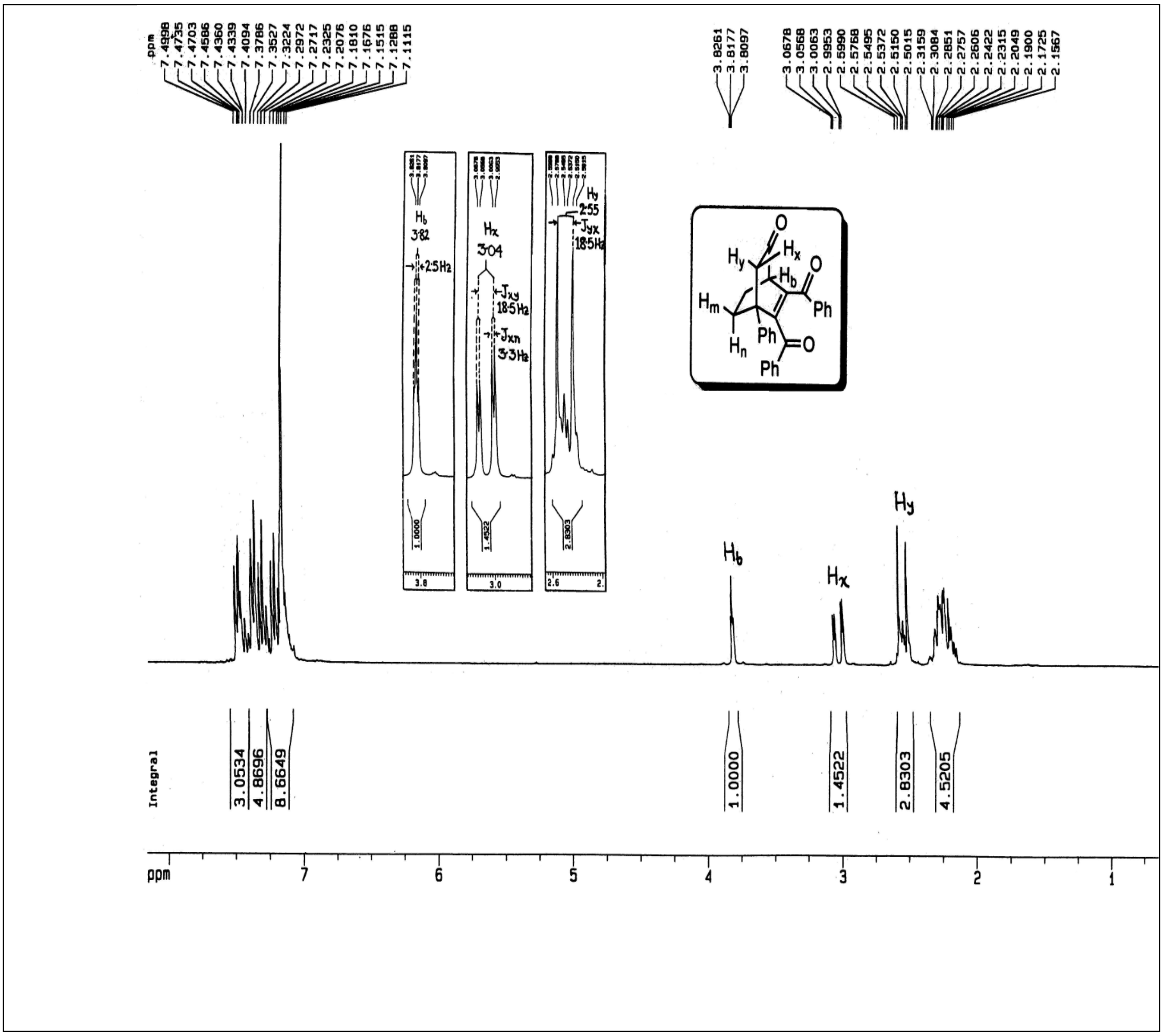

Fig.1: ^{1}H-NMR spectrum of **7a**

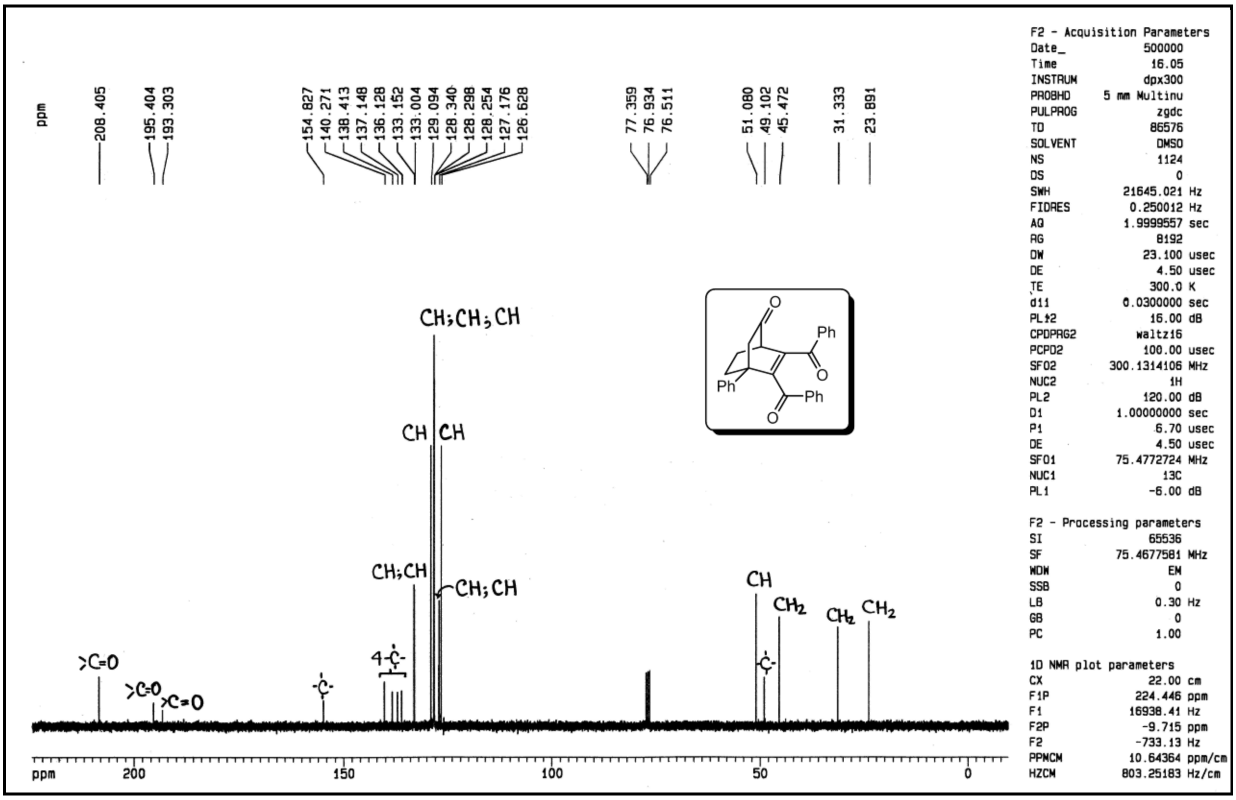

Fig.2: ^{13}C-NMR spectrum of **7a**

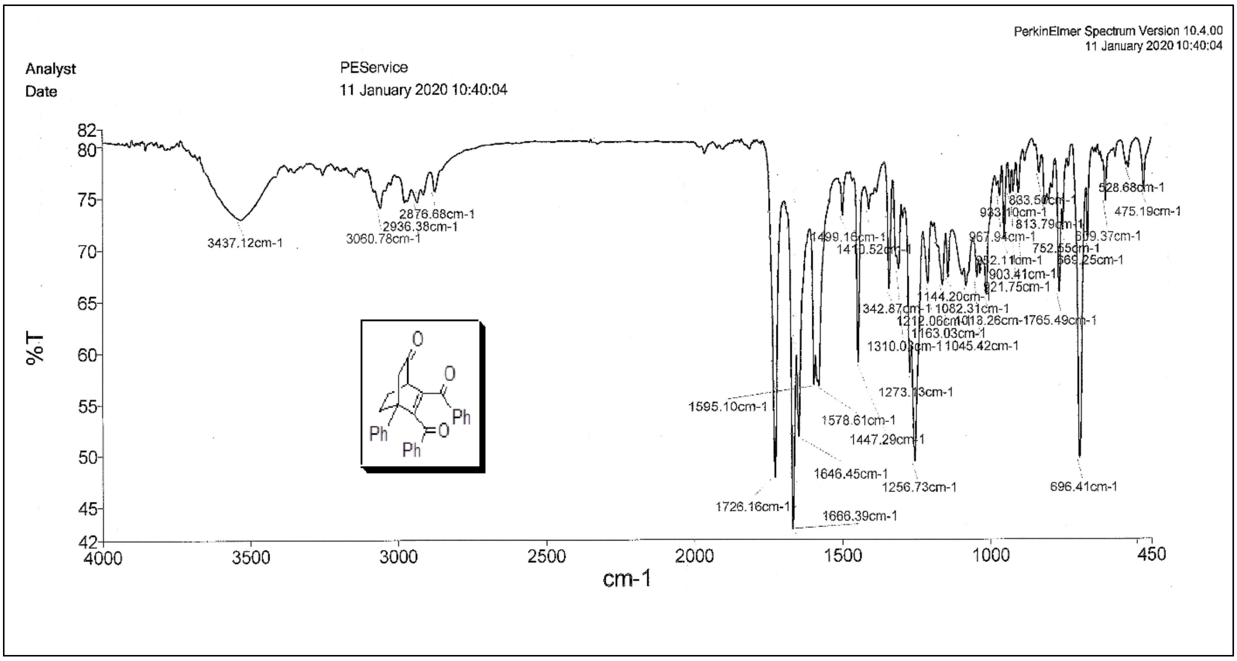

Fig.3: IR spectrum of **7a**

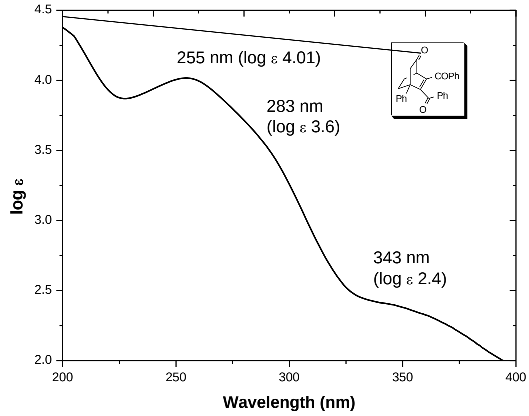

Fig.4: UV absorption spectrum of **7a** in acetonitrile

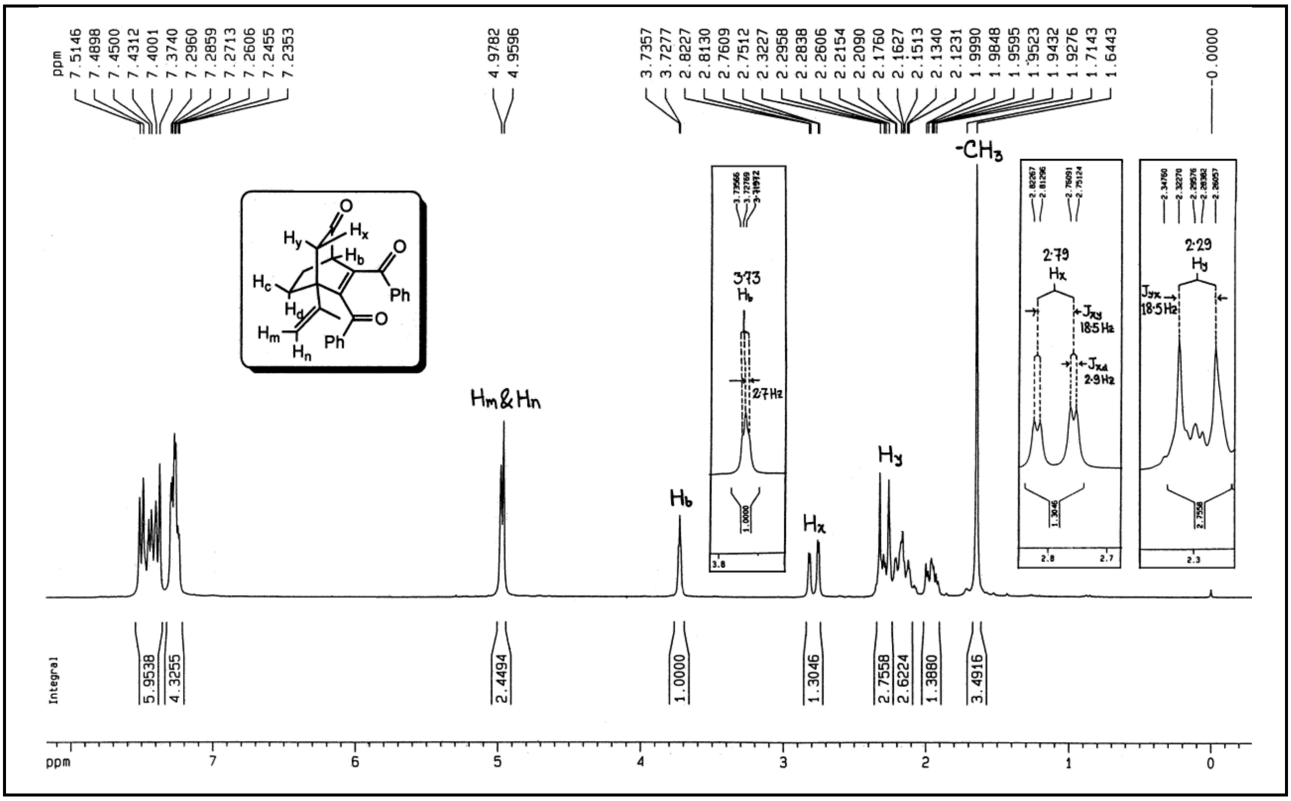

Fig.5: ^{1}H-NMR spectrum of **7b**

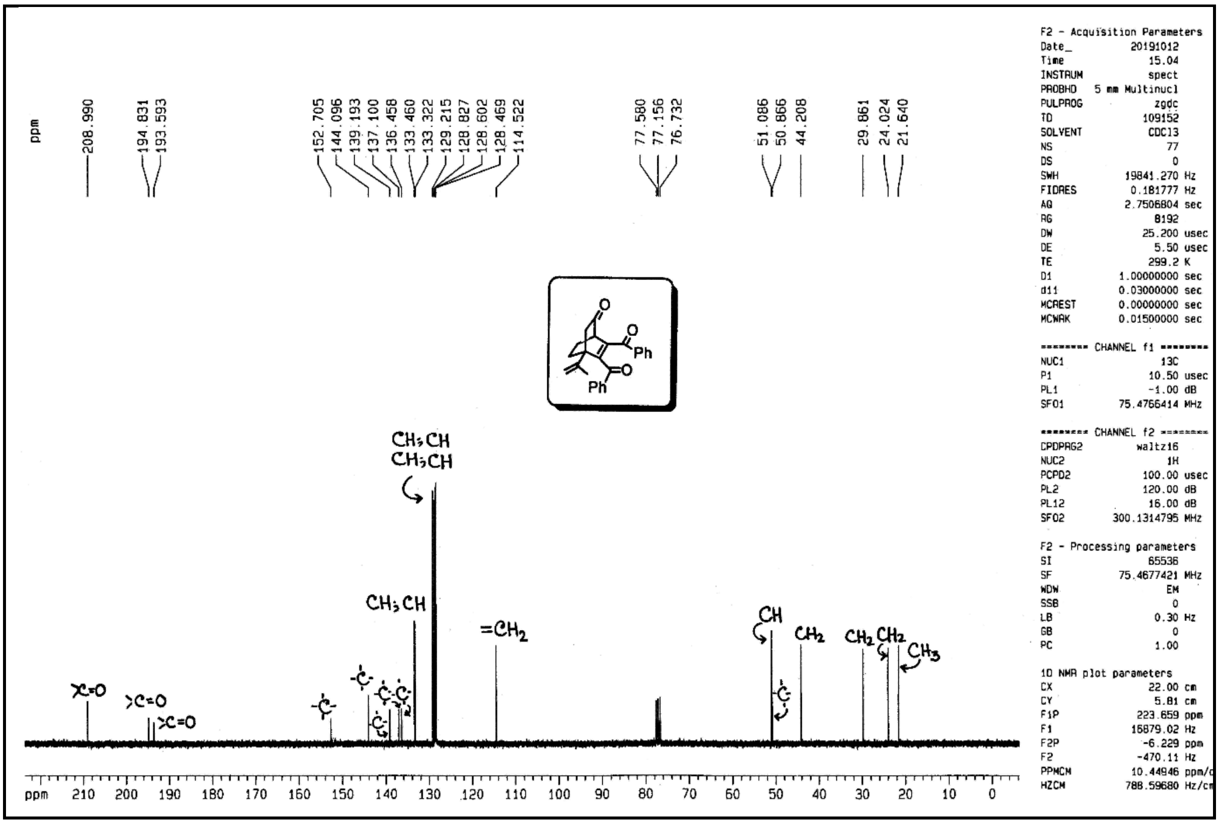

Fig.6: ^{13}C-NMR spectrum of **7b**

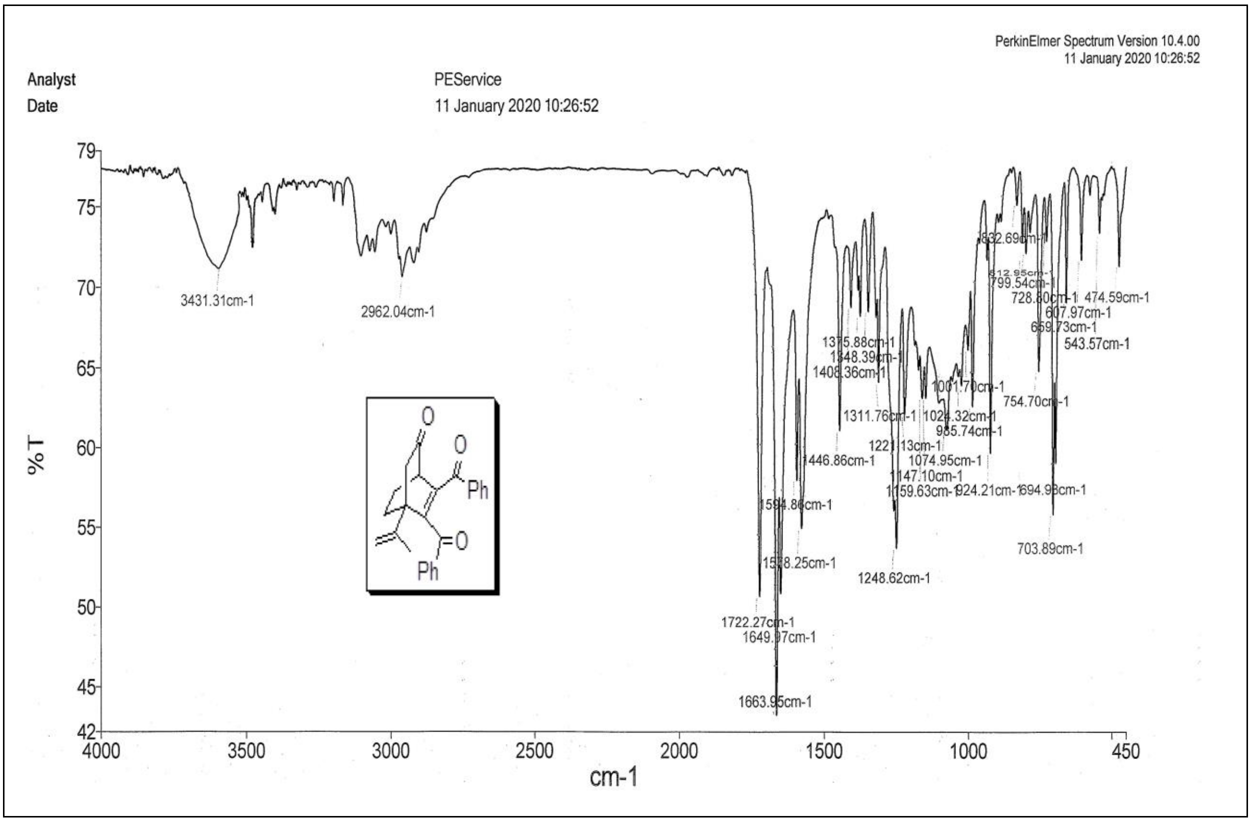

Fig.7: IR spectrum of **7b**

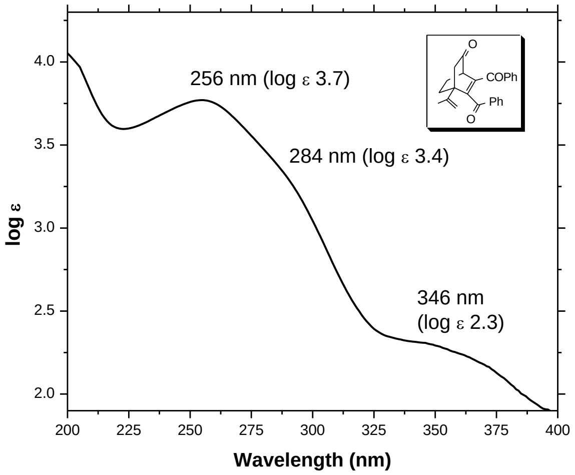

Fig.8: UV absorption spectrum of **7b** in acetonitrile

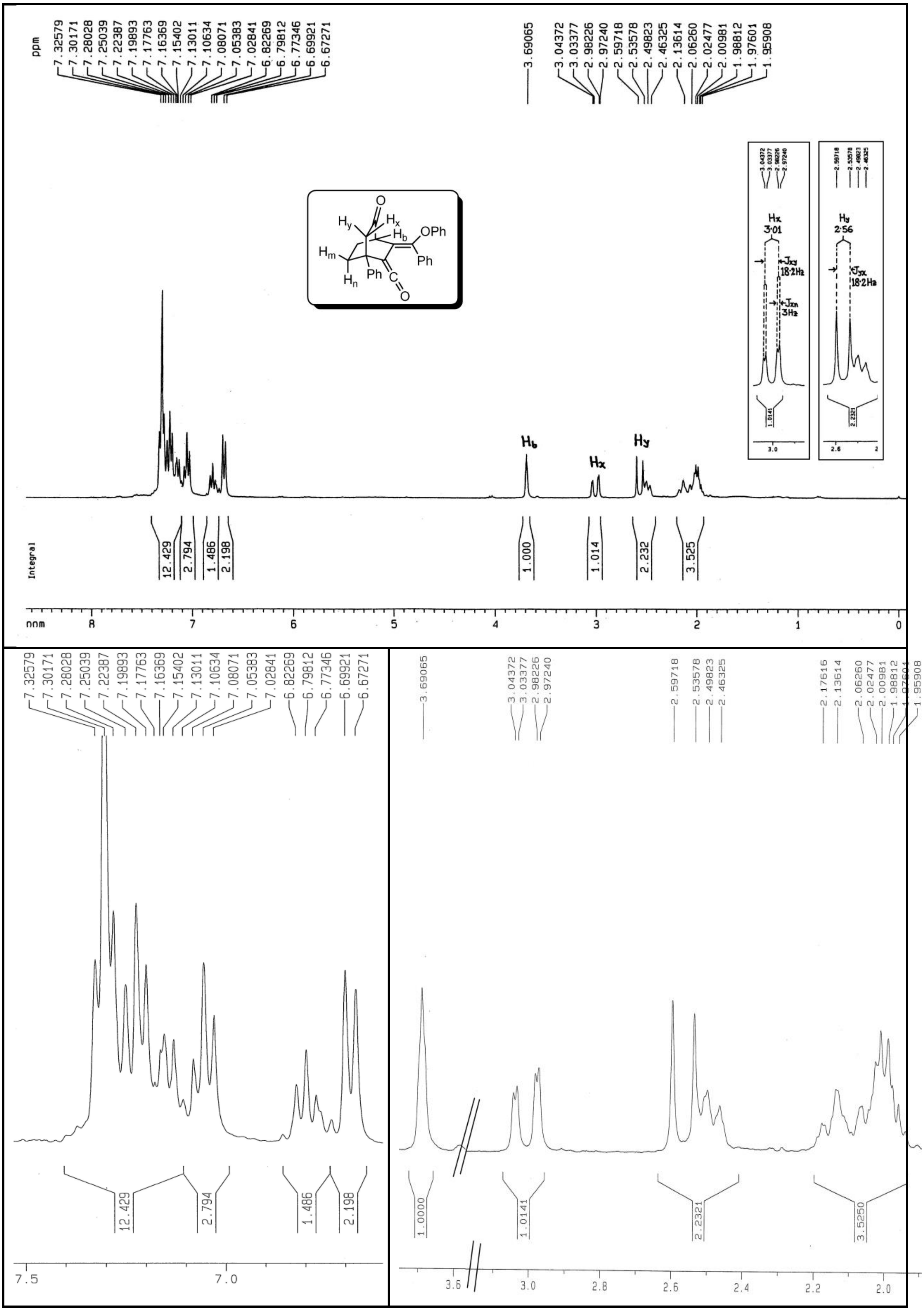

Fig.9: ^{1}H-NMR spectrum of **10a**

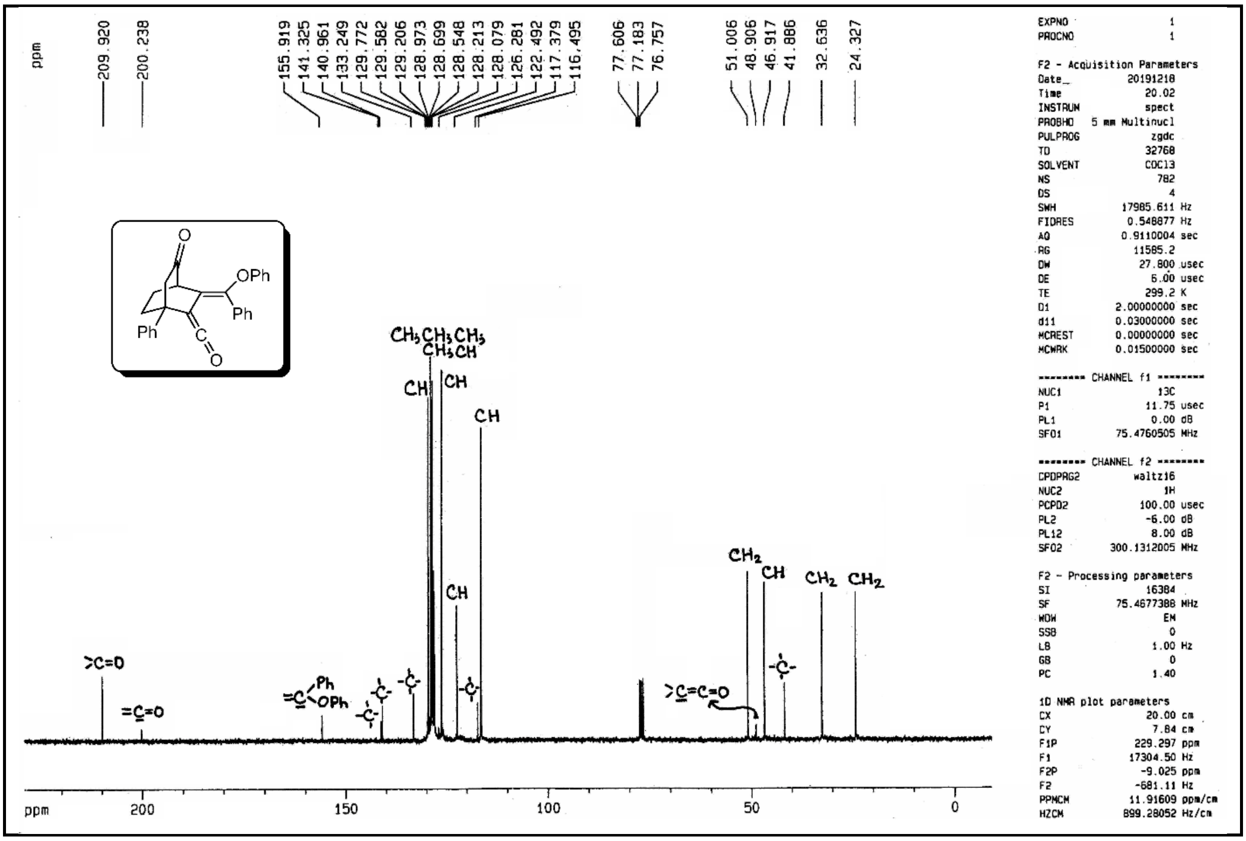

Fig.10: ^{13}C-NMR spectrum of **10a**

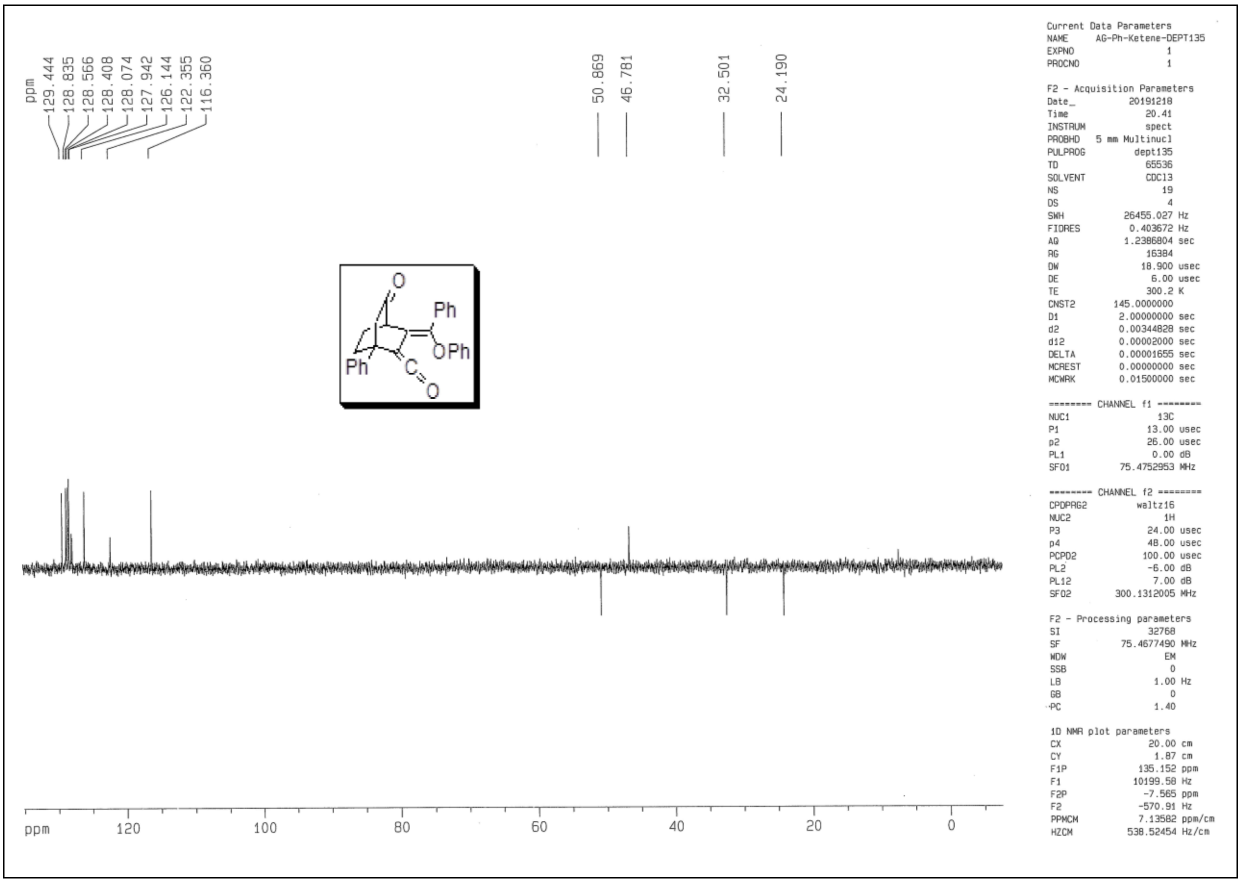

Fig.11: DEPT-135 spectrum of **10a**

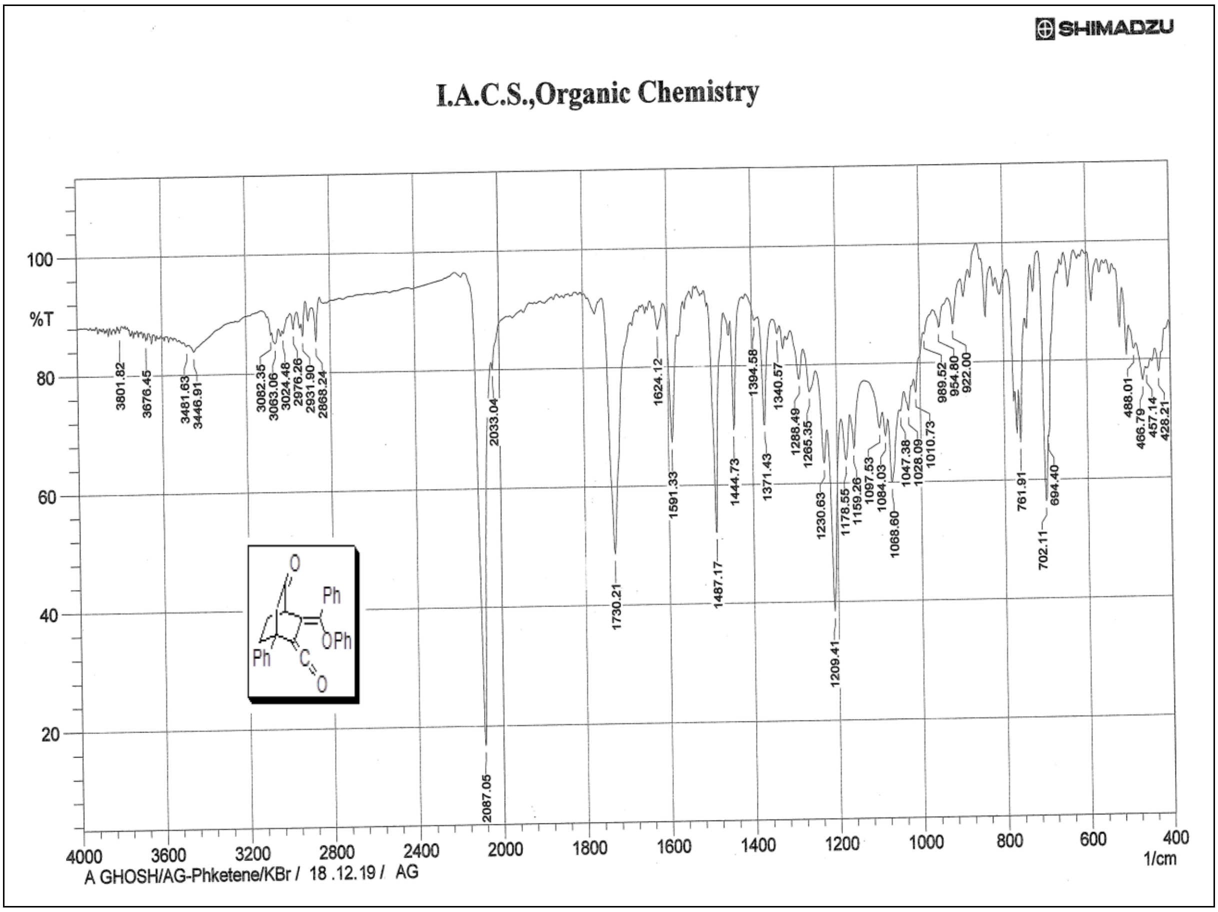

Fig.**12:** IR spectrum of **10a**

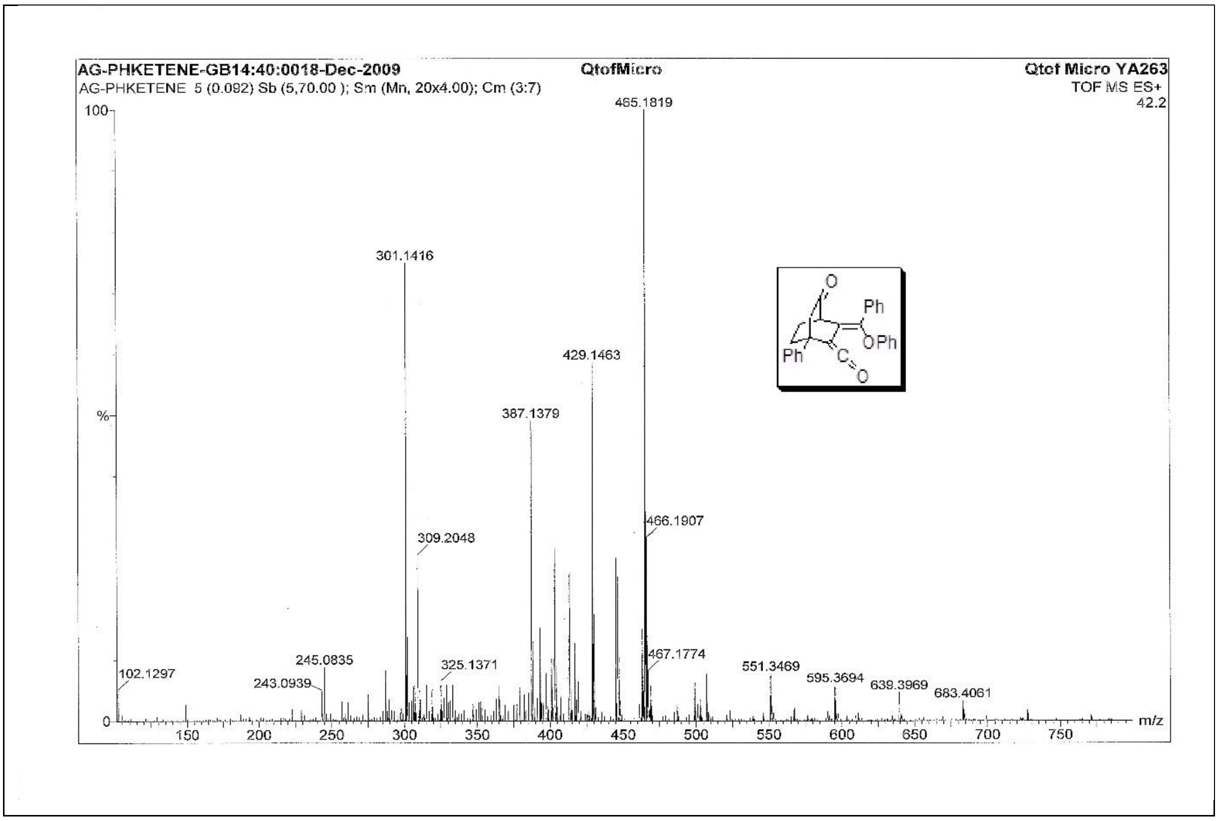

Fig.**13:** Mass spectrum of **10a**

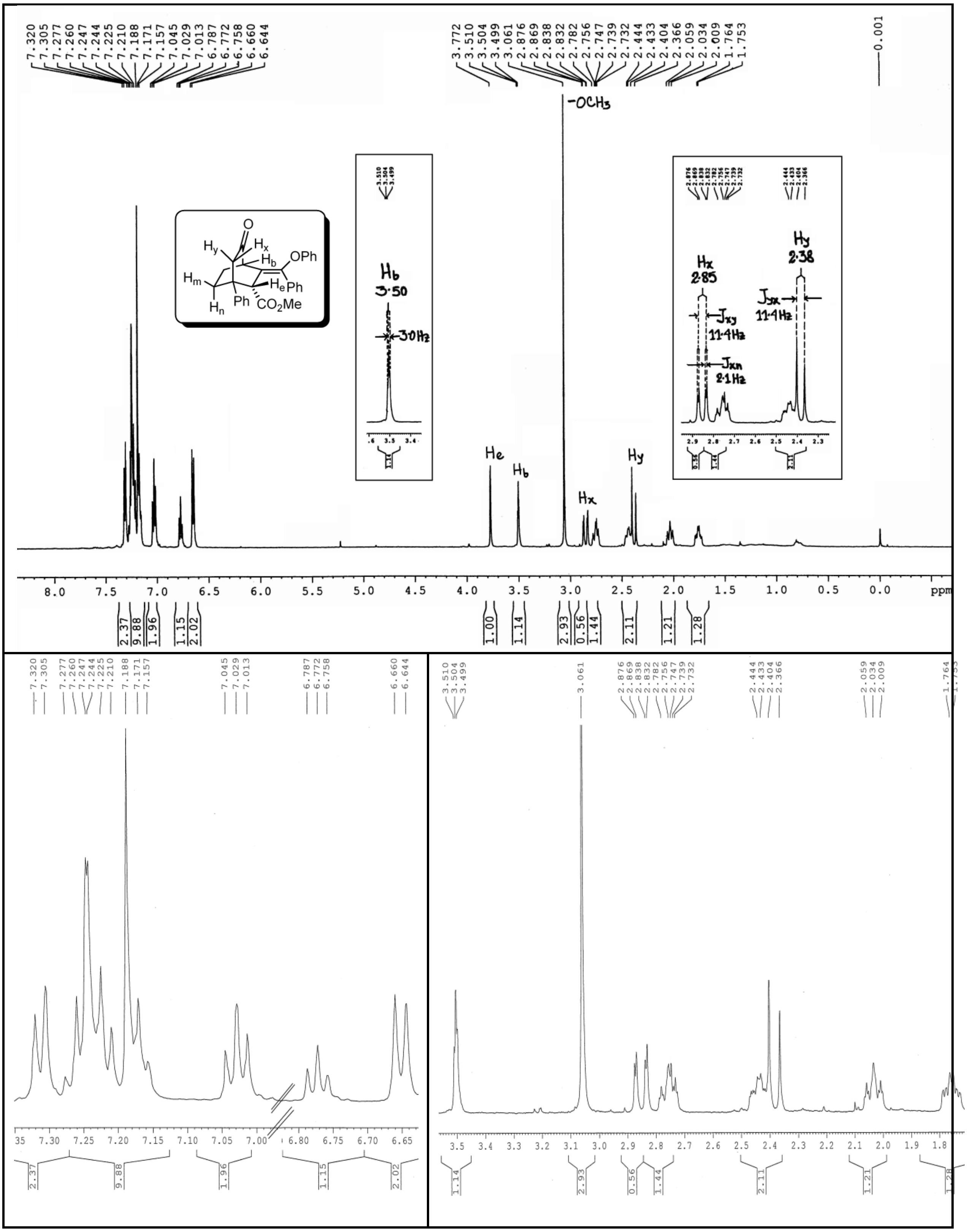

Fig.14: ^{1}H-NMR spectrum of **11a**

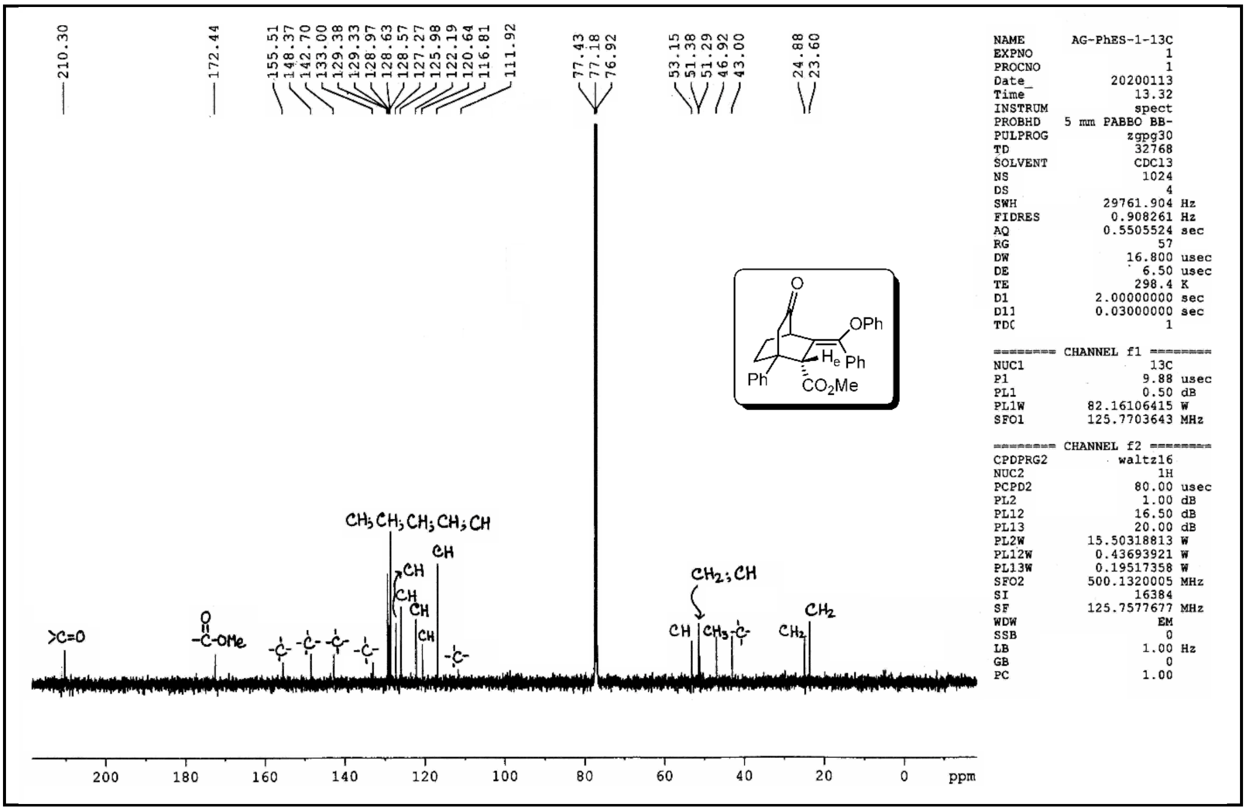

Fig.15: ^{13}C-NMR spectrum of **11a**

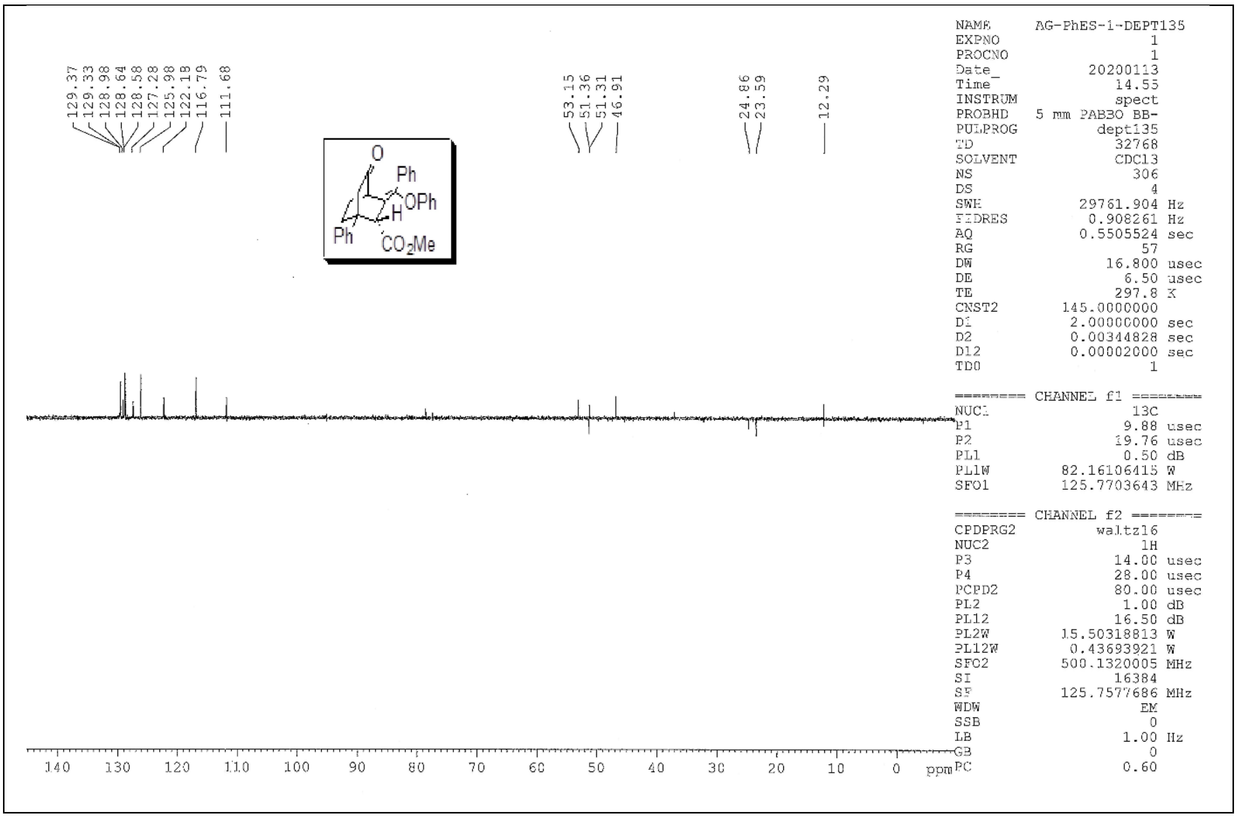

Fig.16: DEPT-135 spectrum of **11a**

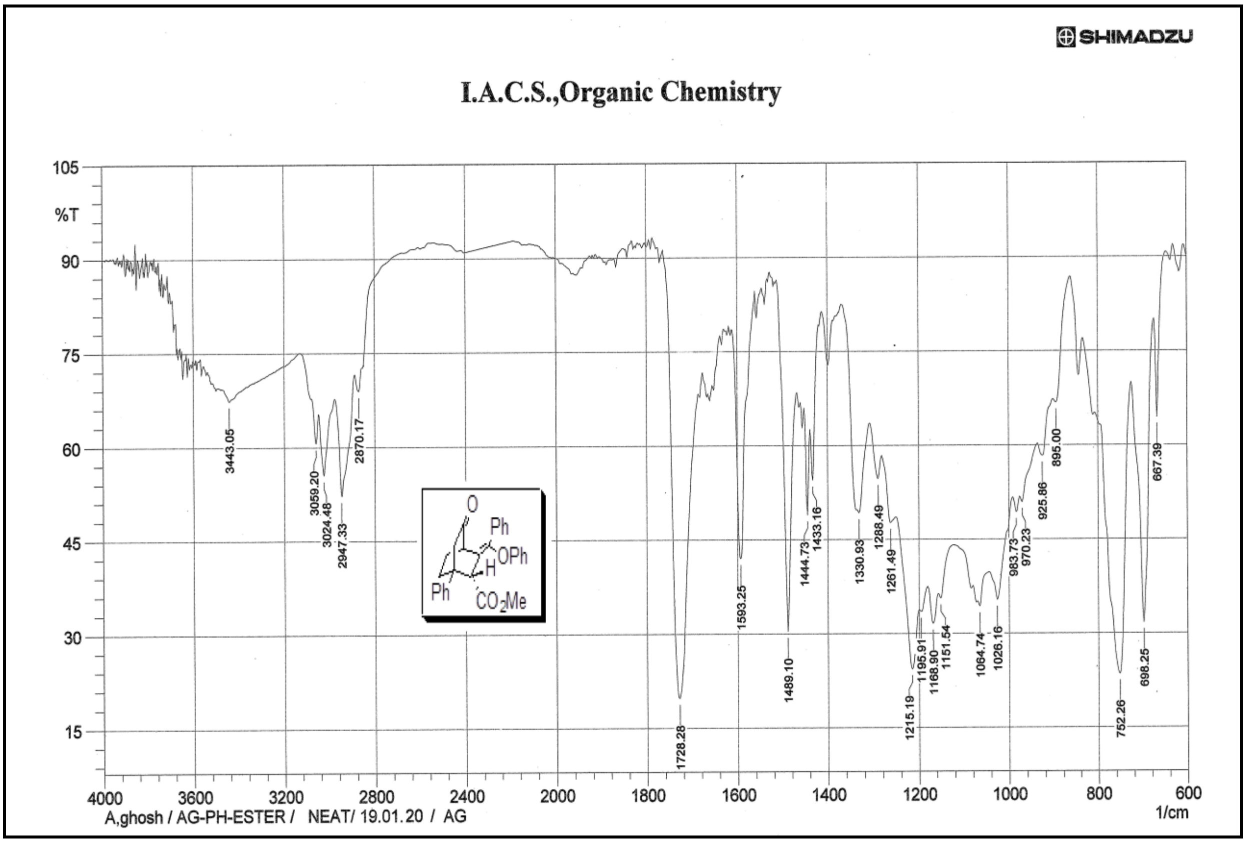

Fig.17: IR spectrum of **11a**

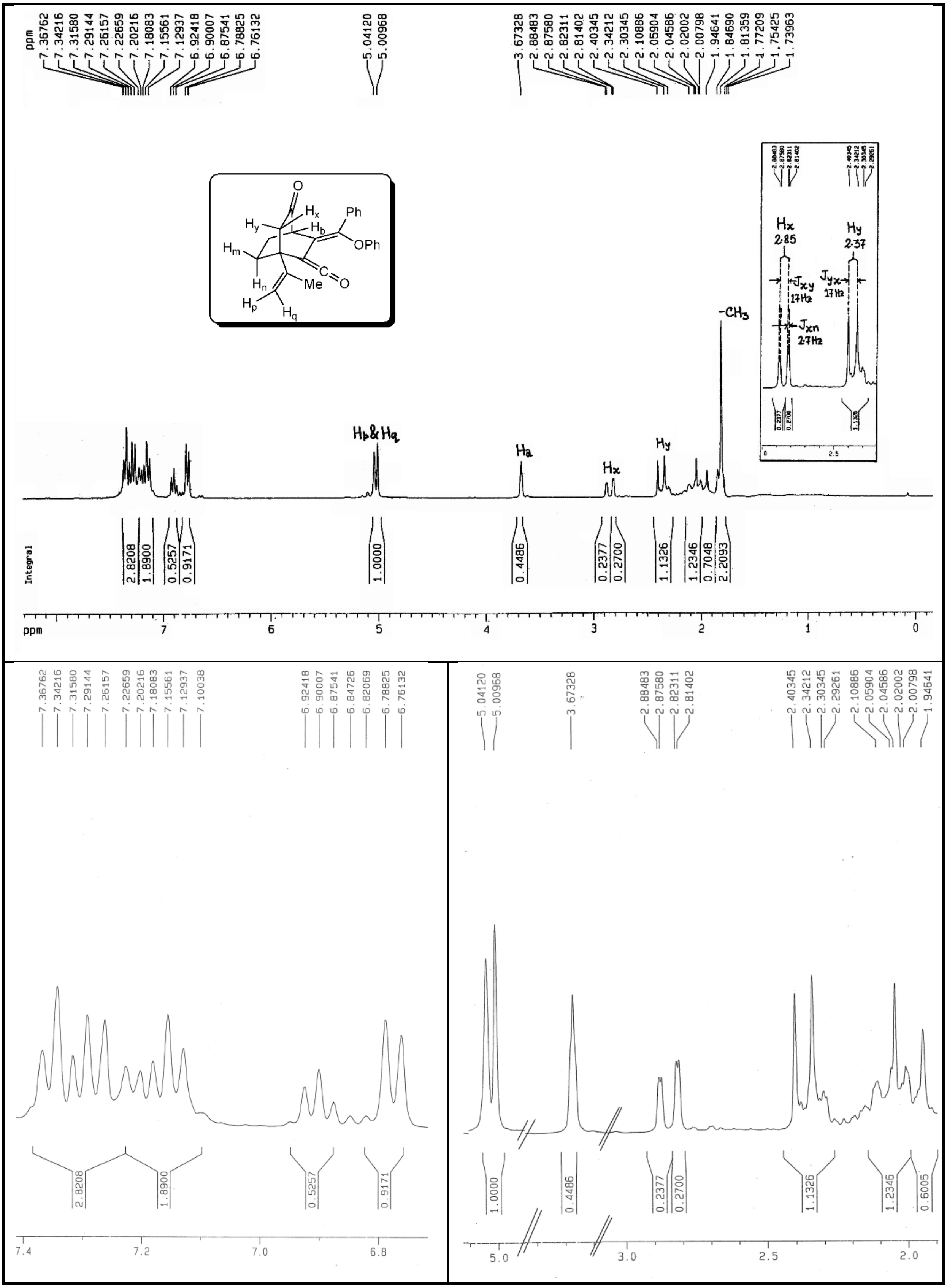

Fig.18: ^{1}H-NMR spectrum of **10b**

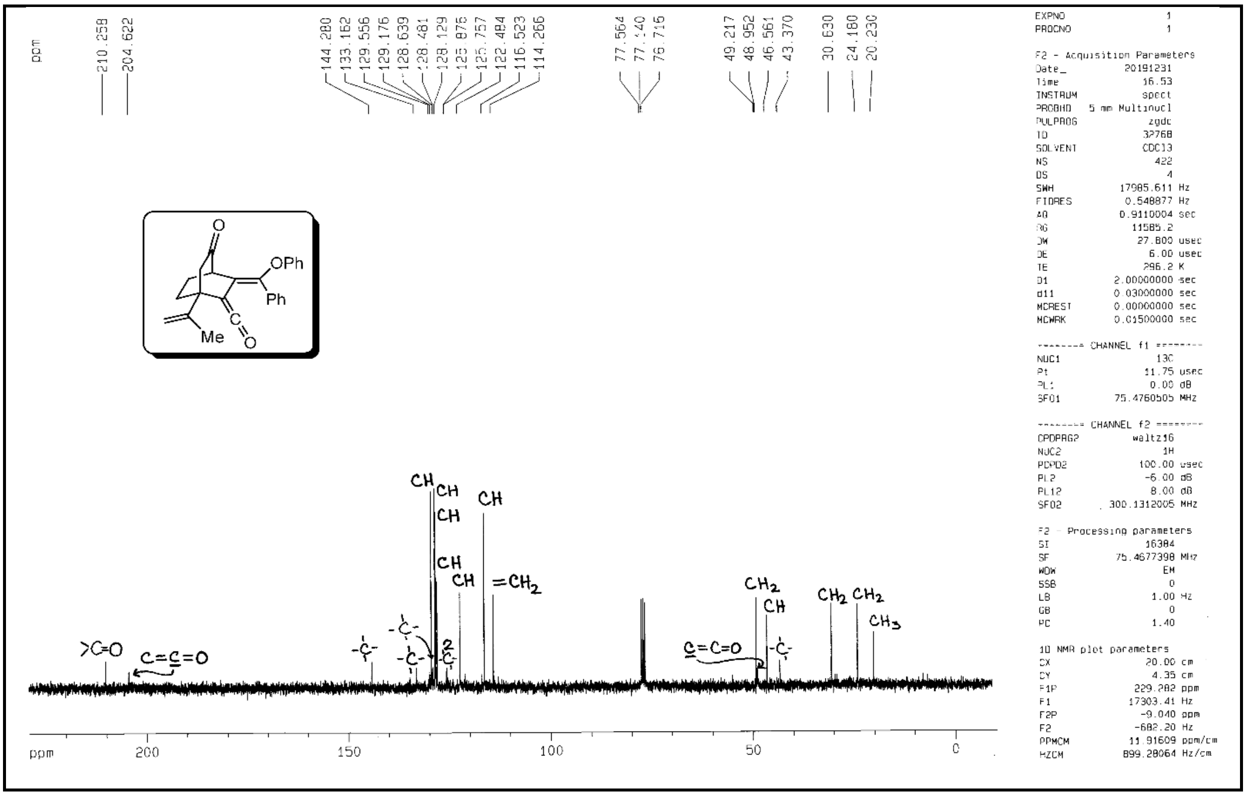

Fig.19: ^{13}C-NMR spectrum of **10b**

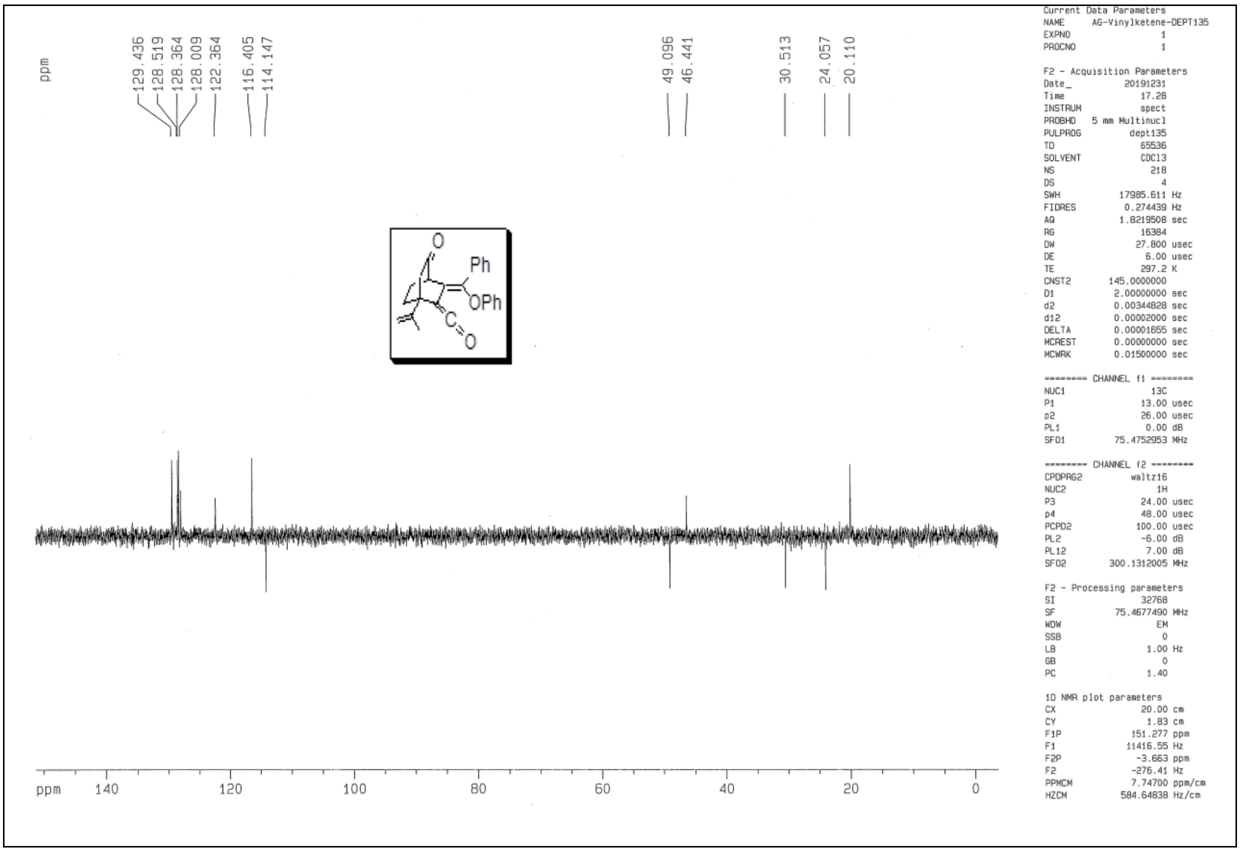

Fig.20: DEPT-135 spectrum of **10b**

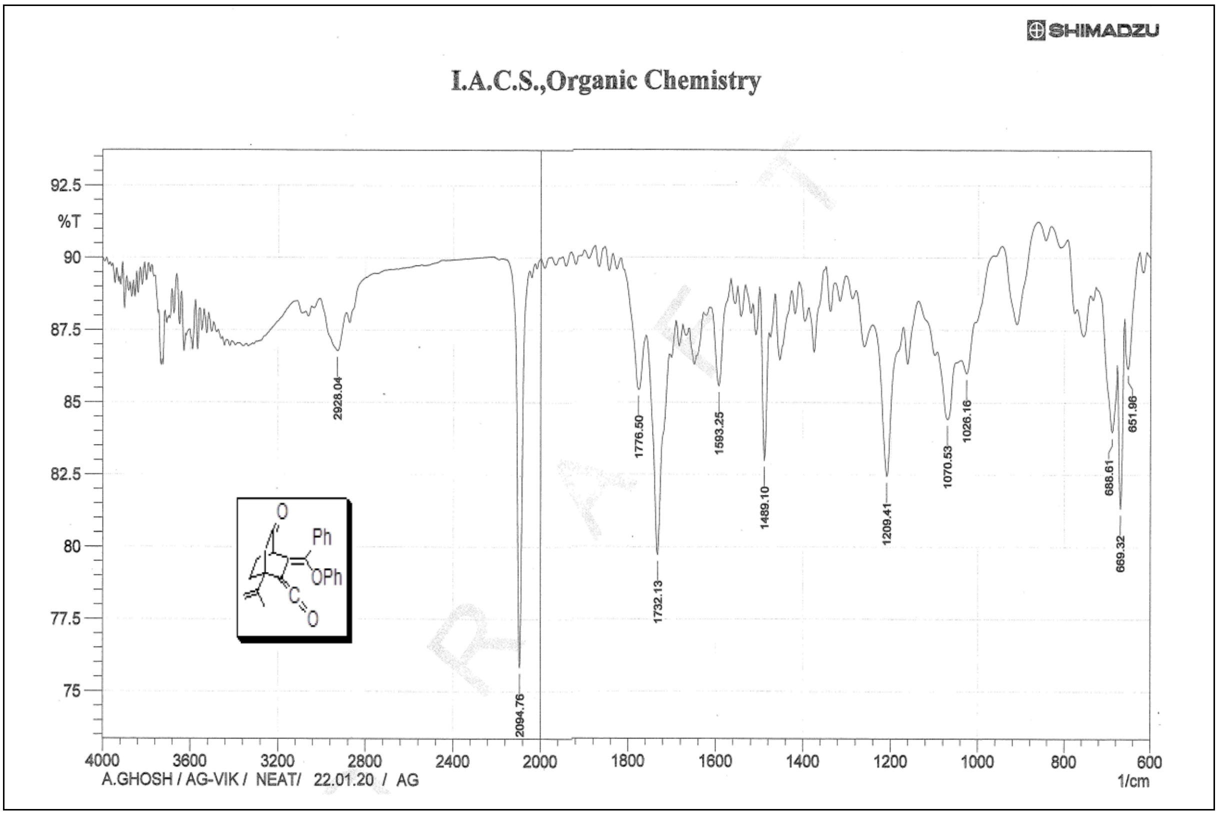

Fig.**21:** IR spectrum of **10b**

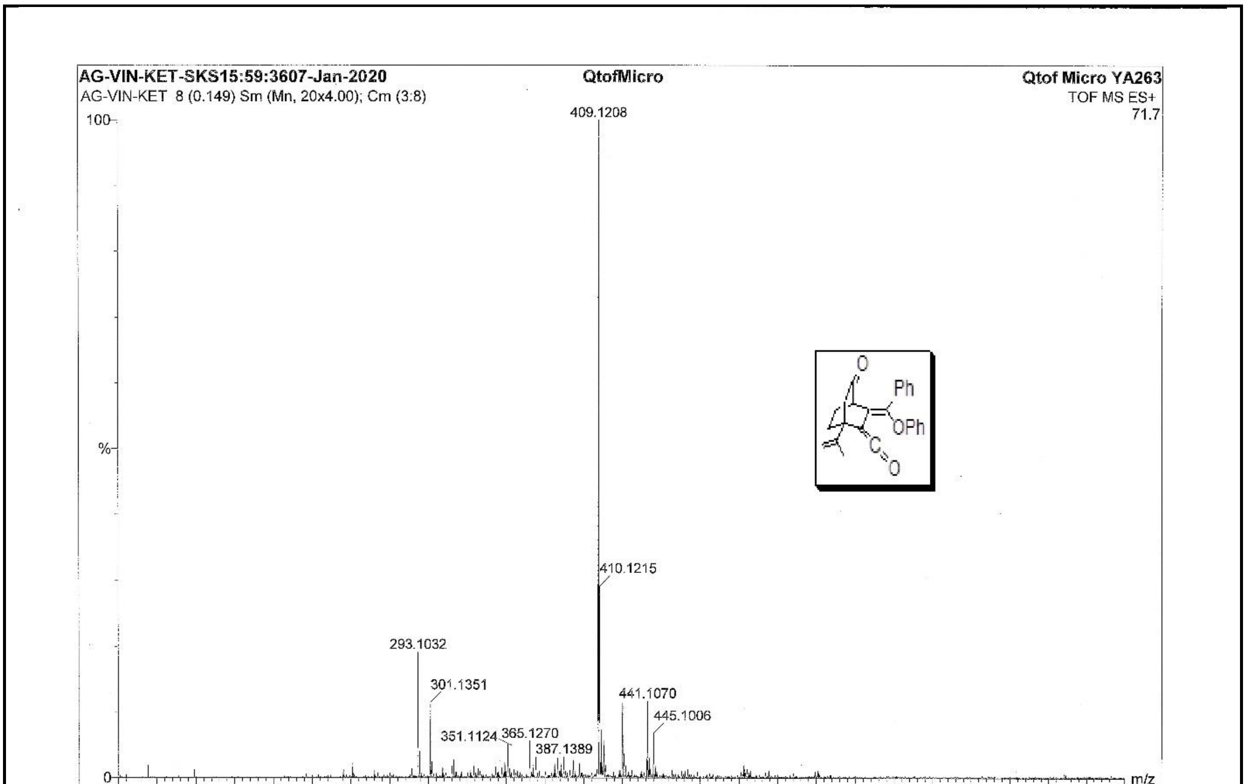

Fig.22: Mass spectrum of **10b**

2-2. ¹H NMR spectra of the crude reaction mixture after photolysis

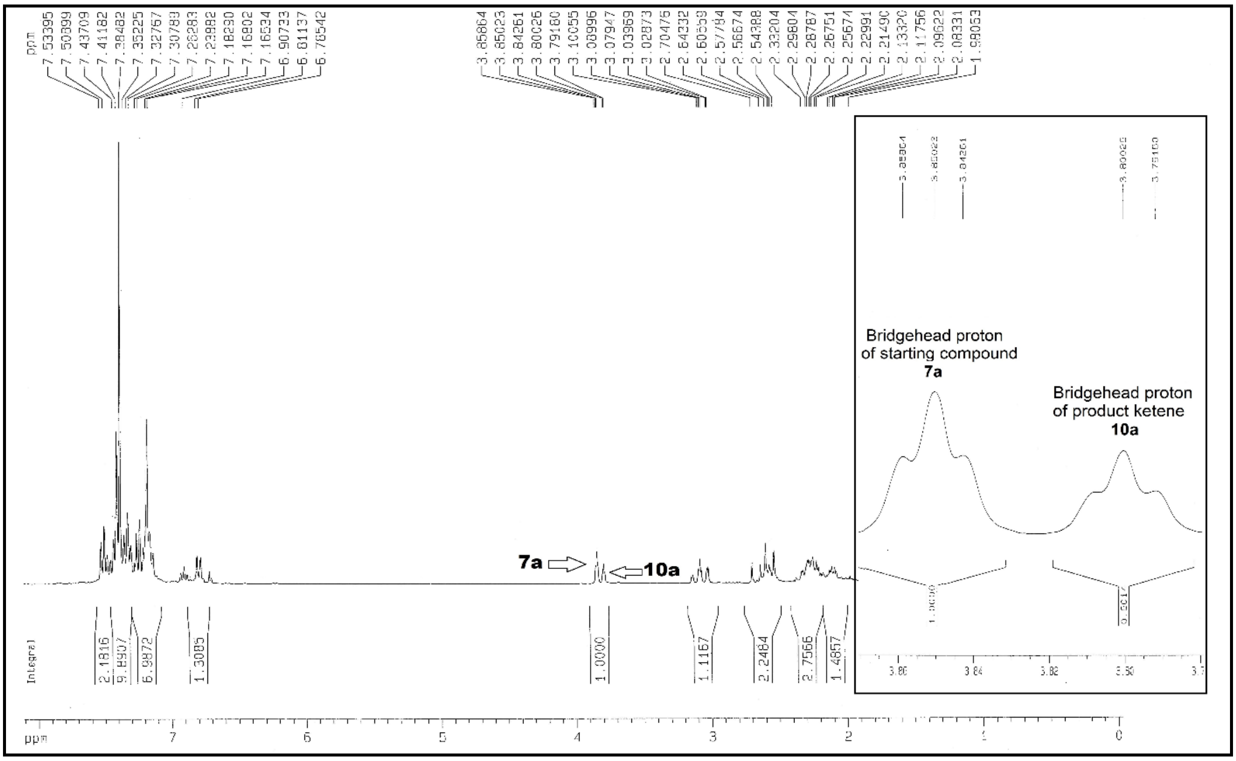

Fig.23: ¹H-NMR spectrum of the crude reaction mixture from the photolysis (at 254 nm wavelength) of **7a** in benzene after 3 h

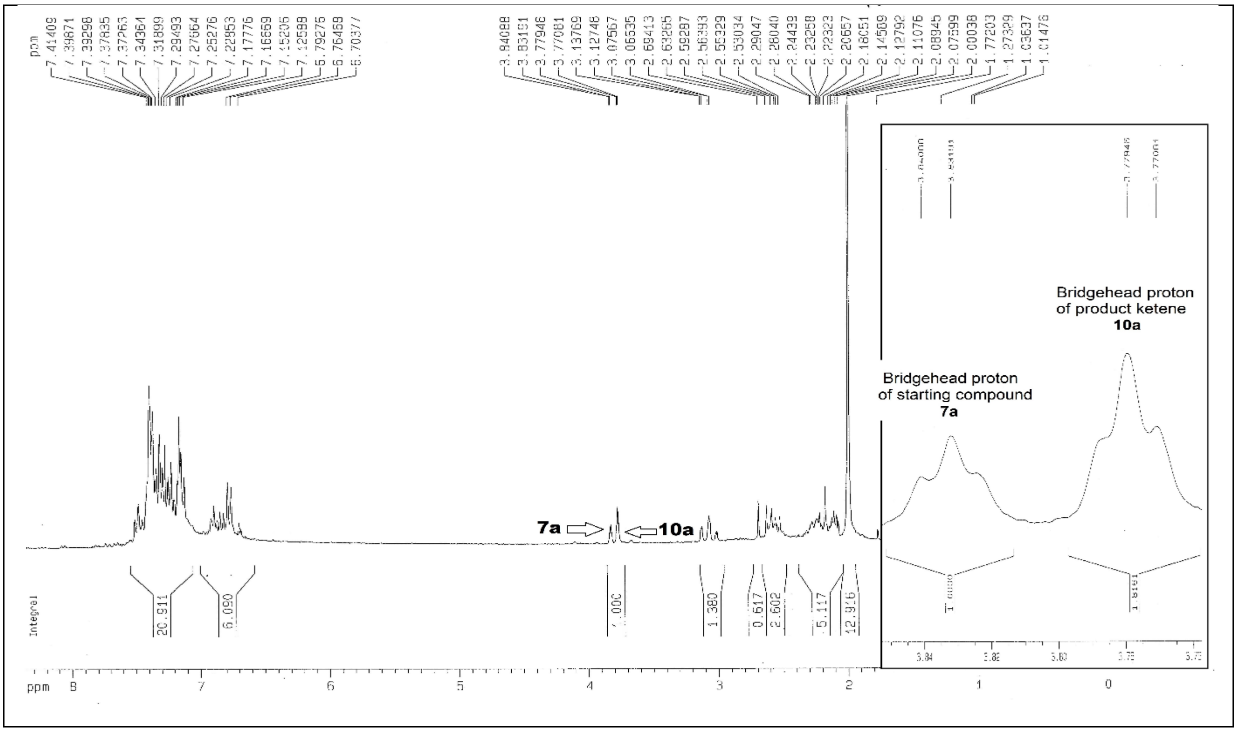

Fig.24: ¹H-NMR spectrum of the crude reaction mixture from the photolysis (at 254 nm wavelength) of **7a** in acetonitrile after 3 h

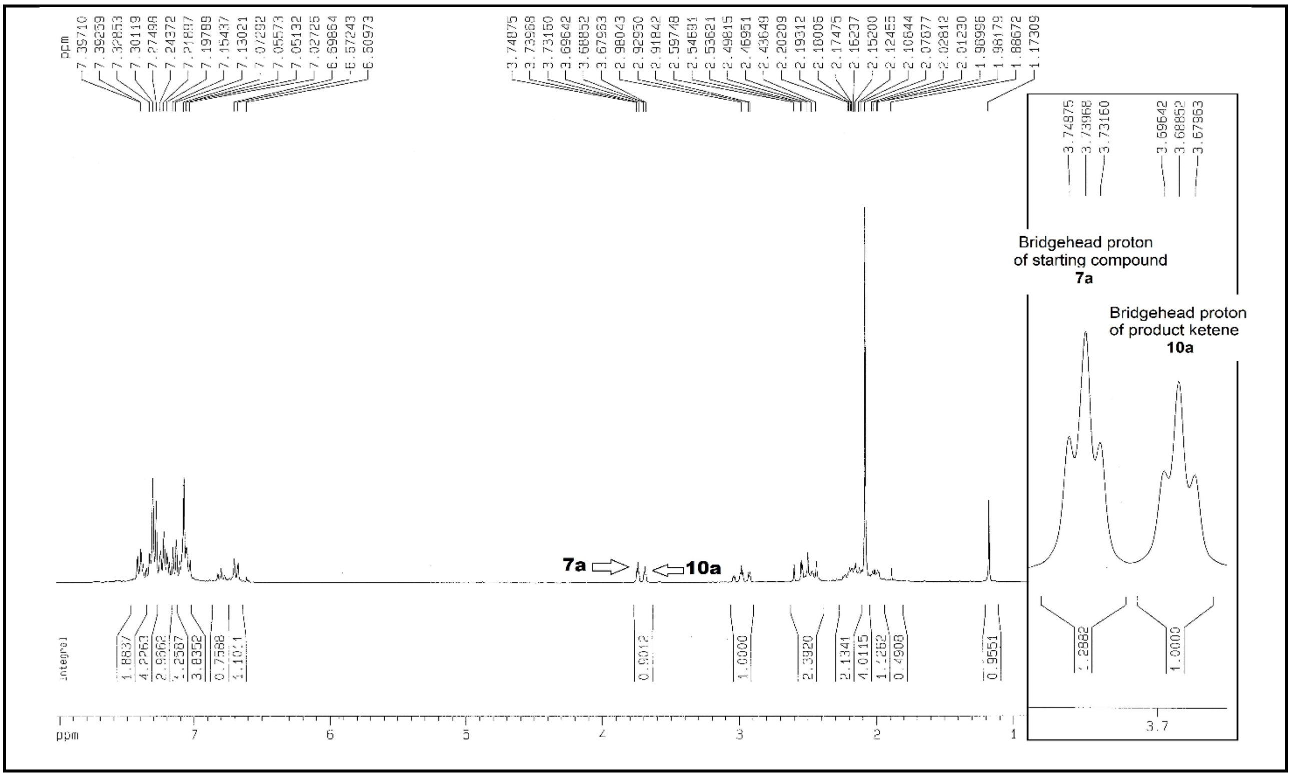

Fig.25: ^{1}H-NMR spectrum of the crude reaction mixture from the photolysis (at 254 nm wavelength) of **7a** in acetone after 3 h

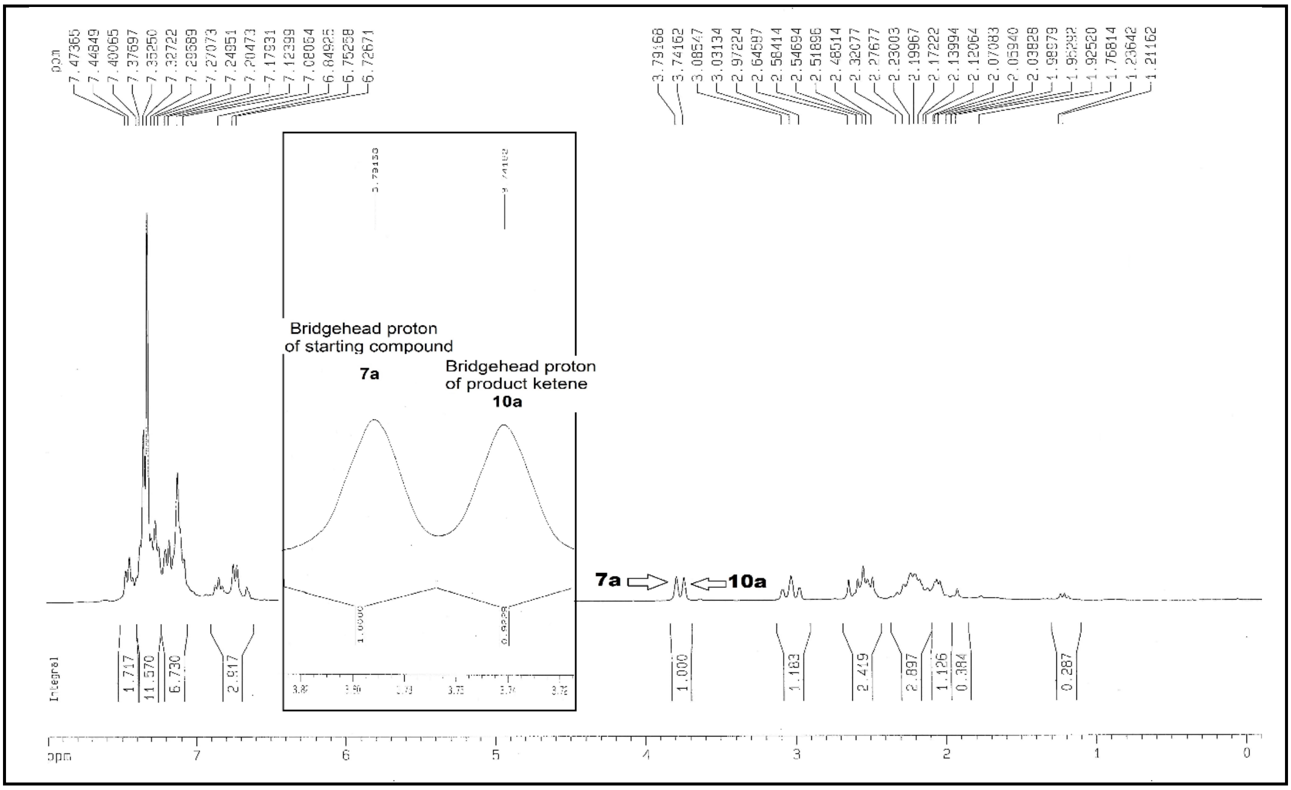

Fig.26: ^{1}H-NMR spectrum of the crude reaction mixture from the photolysis (at 300 nm wavelength) of **7a** in benzene after 4 h

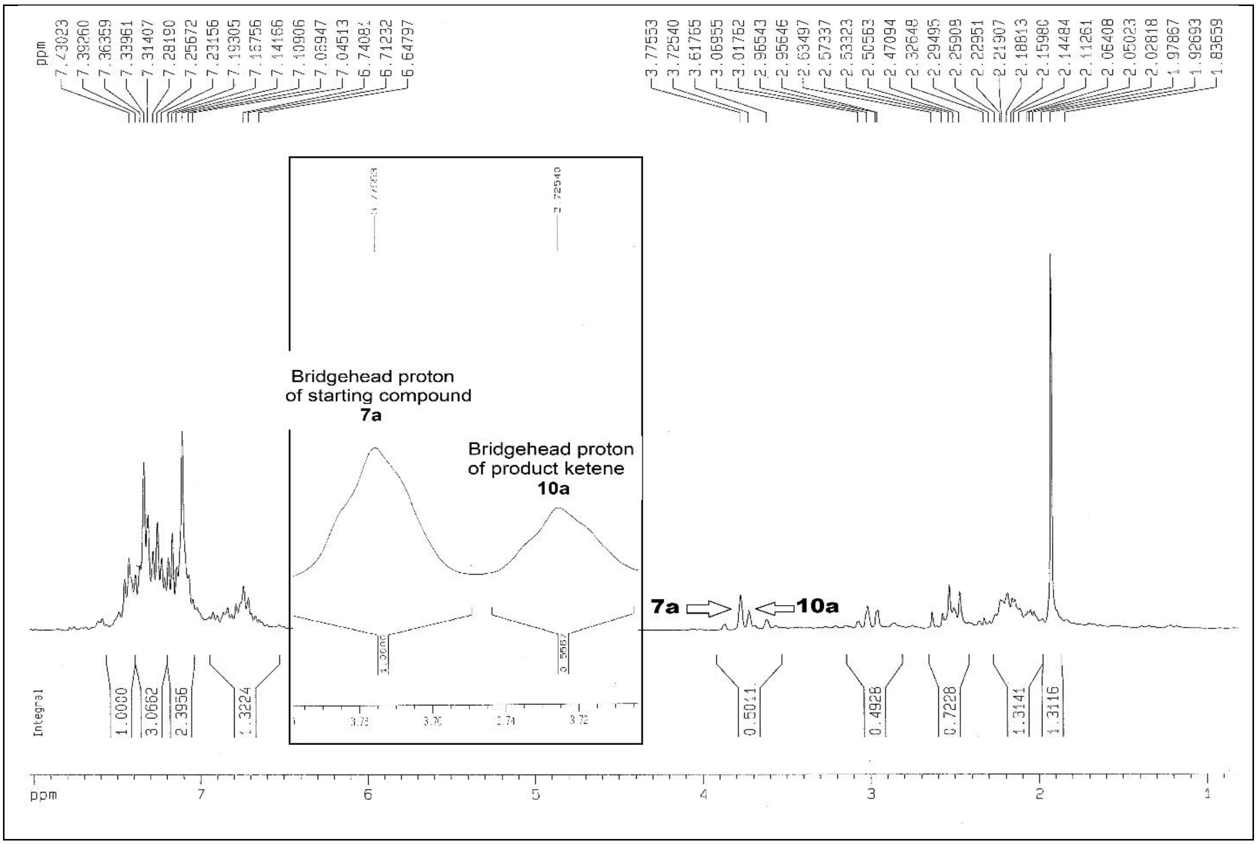

Fig.27: ^{1}H-NMR spectrum of the crude reaction mixture from the photolysis (at 300 nm wavelength) of **7a** in acetonitrile after 4 h

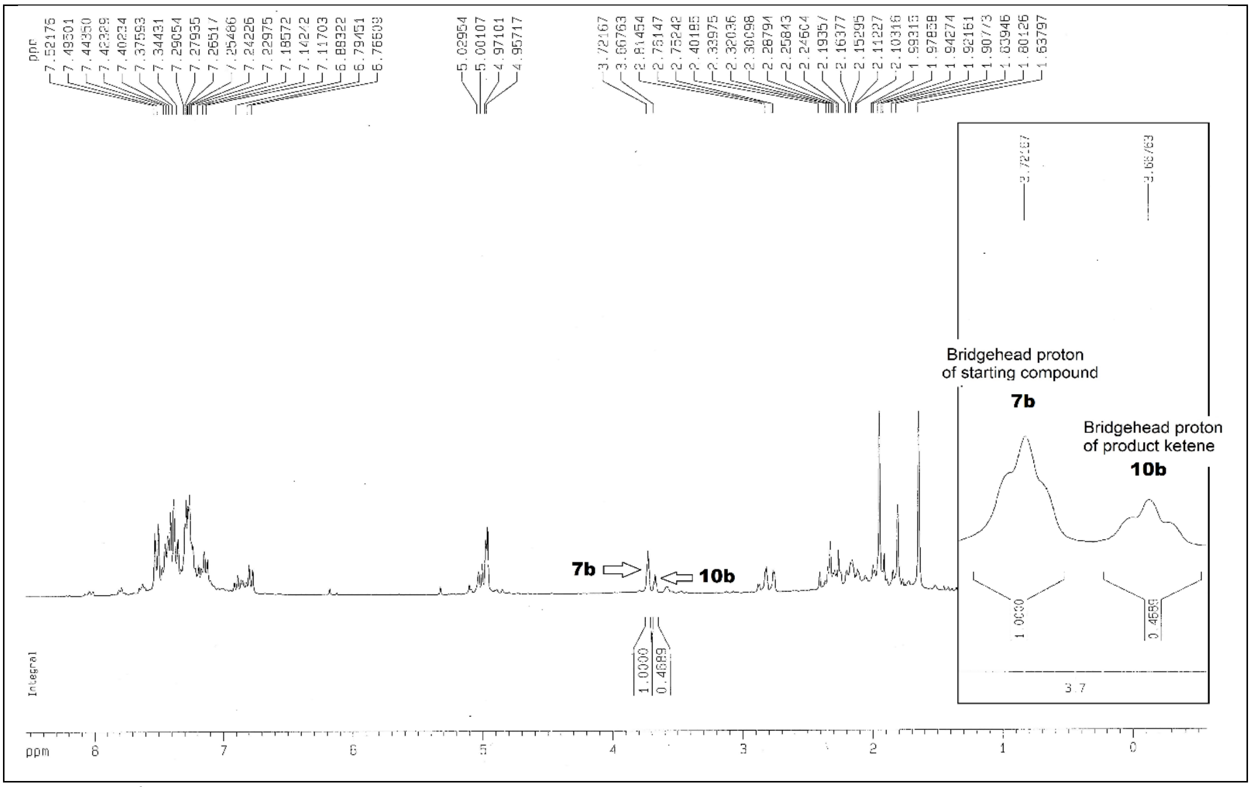

Fig.28: ^{1}H-NMR spectrum of the crude reaction mixture from the photolysis (at 254 nm wavelength) of **7b** in benzene after 2.5 h
